# The conserved basic residues and the charged amino acid residues at the α-helix of the zinc finger motif regulate the nuclear transport activity of triple C_2_H_2_ zinc finger proteins

**DOI:** 10.1371/journal.pone.0191971

**Published:** 2018-01-30

**Authors:** Chih-Ying Lin, Lih-Yuan Lin

**Affiliations:** Institute of Molecular and Cellular Biology and Department of Life Science, National Tsing Hua University, Hsinchu, Taiwan, ROC; Imperial College London, UNITED KINGDOM

## Abstract

Zinc finger (ZF) motifs on proteins are frequently recognized as a structure for DNA binding. Accumulated reports indicate that ZF motifs contain nuclear localization signal (NLS) to facilitate the transport of ZF proteins into nucleus. We investigated the critical factors that facilitate the nuclear transport of triple C_2_H_2_ ZF proteins. Three conserved basic residues (hot spots) were identified among the ZF sequences of triple C_2_H_2_ ZF proteins that reportedly have NLS function. Additional basic residues can be found on the α-helix of the ZFs. Using the ZF domain (ZFD) of Egr-1 as a template, various mutants were constructed and expressed in cells. The nuclear transport activity of various mutants was estimated by analyzing the proportion of protein localized in the nucleus. Mutation at any hot spot of the Egr-1 ZFs reduced the nuclear transport activity. Changes of the basic residues at the α-helical region of the second ZF (ZF2) of the Egr-1 ZFD abolished the NLS activity. However, this activity can be restored by substituting the acidic residues at the homologous positions of ZF1 or ZF3 with basic residues. The restored activity dropped again when the hot spots at ZF1 or the basic residues in the α-helix of ZF3 were mutated. The variations in nuclear transport activity are linked directly to the binding activity of the ZF proteins with importins. This study was extended to other triple C_2_H_2_ ZF proteins. SP1 and KLF families, similar to Egr-1, have charged amino acid residues at the second (α2) and the third (α3) positions of the α-helix. Replacing the amino acids at α2 and α3 with acidic residues reduced the NLS activity of the SP1 and KLF6 ZFD. The reduced activity can be restored by substituting the α3 with histidine at any SP1 and KLF6 ZFD. The results show again the interchangeable role of ZFs and charge residues in the α-helix in regulating the NLS activity of triple C_2_H_2_ ZF proteins.

## Introduction

Zinc finger domain (ZFD) is among the most common DNA binding structures found in eukaryotic transcription factors. A variety of ZF motifs have been identified [1–3]. The most typical structure has two cysteines and two histidines (C_2_H_2_) to interact with a zinc ion. Approximately 700 genes encoding C_2_H_2_ ZFD have been identified in the human genome. They represent the most abundant DNA binding proteins in cells [4]. C_2_H_2_ ZFD was identified initially in the transcription factor TFIIIA of *Xenopus laevis* [5] and presumed to present only in eukaryotic cells. Later studies indicate that proteins with C_2_H_2_ ZFD also exist in prokaryotic cells, suggesting a structural conservation in evolution [6]. Besides DNA binding, the C_2_H_2_ motif of the ZF proteins participates also in RNA binding [7, 8] and protein-protein interactions [9].

The C_2_H_2_ ZF motif has a highly conserved structure. It contains 28–30 amino acid residues with a consensus sequence of ψ-X-C-X_2-4_-C-X_3_-ψ-X_5_-ψ-X_2_-H-X_3-4_-H (ψ stands for hydrophobic and X represents any amino acid residues) which folds into a ßßα secondary structure. The zinc ion binds to the cysteine and histidine residues to form a unique steric configuration [10–13]. The α-helix of the motif inserts into the major groove of DNA. Each C_2_H_2_ ZF motif covers three base pairs of DNA generating a specific recognition between the ZF protein and a DNA sequence [14, 15].

ZF proteins usually have tandem repeats of ZFs connected by a consensus Thr-Gly-Glu-Lys-Pro sequence. Based on the number and the distribution of the C_2_H_2_ ZF, proteins can be categorized as (a) single, (b) triple, (c) multiple adjacent and (d) separated paired C_2_H_2_ ZF proteins [16]. The C_2_H_2_ motif on the single C_2_H_2_ ZF protein has to coordinate with other region of the protein for DNA binding [17]. The other types of C_2_H_2_ ZF proteins can interact directly with DNA. For triple C_2_H_2_ ZF proteins, such as Egr-1 (Zif268), Krüppel-like Factor (KLF) or SP1, all the ZF domains are involved in DNA binding and even protein-protein interaction [18–20]. However, the role of ZF may vary in multiple adjacent C_2_H_2_ ZF proteins. For example, ZF1-ZF4 of Miz-1 are responsible for interacting with SMAD3 while the rest of the ZF motifs participate in binding the target promoter [21]. OAZ has 30 ZF motifs. Only ZF2-ZF5 interacts with specific DNA sequence, whereas all other ZF motifs can interact with proteins [22]. These findings demonstrate the diverse functions of ZFs.

Proteins are synthesized in the cytoplasm. For transcription factors that localize mainly in the nucleus, they have to be actively transported into the nucleus. These proteins have a requisite nuclear localization signal (NLS) that recognize by carrier proteins (importin or karyopherin) for the process of translocating through the nuclear pore [23, 24]. Classical NLS consists of consecutive basic amino acid residues occur in one (monopartite) [25] or two (bipartite) fragments separated by 10–12 residues [26, 27]. Proteins carrying classical NLS can be recognized by Importinα/ß [28–30]. Importinα (Impα) binds the cargo protein while Importinß (Impß) interacts with Impα. The complex then interacts with nucleoporins at the cytoplasmic side of the nuclear pore complex and transports the cargo protein into the nucleus [31–34].

Recent studies indicate that the C_2_H_2_ ZFD can serve as an NLS to assist the nuclear entrance of ZF proteins. Unlike proteins with the classical NLS that contains consecutive basic residues, the transport mechanism for ZF proteins is not clear. However, the basic residues in the C_2_H_2_ ZF motifs are considered to associate functionally with nuclear transport. Noticeably, most proteins with a single C_2_H_2_ ZF motif have difficulty in translocating into the nucleus. At least two C_2_H_2_ ZF motifs are required in a ZFD to serve as NLS [35–40]. Evidence from recent studies suggest that residues from a few adjacent ZF motifs of a triple or multiple C_2_H_2_ ZF protein [36, 41–45] can serve as NLS. Specific structural characteristics and/or residues in these ZF motifs are recognized by the carrier proteins [42, 46]. Besides binding with Impα [43, 47], C_2_H_2_ ZF proteins can bind directly with Impβ or β-like importins for transport [35, 48]. C_2_H_2_ ZF motifs are highly conserved in structure and even basic amino acid residues distribution. Since the number and the role of C_2_H_2_ ZF vary in ZF proteins, the function of each ZF cannot be distinguished easily. In this study, we covalently joined the ZFD of triple C_2_H_2_ ZF proteins to green fluorescent proteins (GFP) and investigated the nuclear transport activity of the fusion proteins. Residues on the ZFD that modulate the nuclear transport process are characterized.

## Materials and methods

### Cell culture and chemicals

Chinese hamster ovary (CHO) K1 cells (ATCC^®^ CCL-61^™^) were cultured at 37 °C in McCoy's 5A medium with 10% heat-inactivated fetal bovine serum (FBS), 0.22% sodium bicarbonate, 100 units/ml ampicillin, and 100 mg/ml streptomycin in 5% CO_2_, 95% air and 100% humidity. Reagent grade chemicals were purchased from Sigma unless specified. Cell culture reagents were purchased from Invitrogen/Gibco. DNA polymerase (KOD-plus) was obtained from TOYOBO. Lipofectamine^™^ 2000 was obtained from Invitrogen. TALON^®^ Metal Affinity resin was provided by Clontech. Glutathione Sepharose 4B beads and secondary antibodies (anti-mouse and anti-rabbit IgGs) were from GE Healthcare. Protein and DNA molecular weight markers were from SMOBIO. Anti-GFP (ab290) antibodies were purchased from Abcam. Anti-His (C-term) and anti-GST (sc-138) antibodies were from Invitrogen and Santa Cruz Biotechnology, respectively.

### Plasmid constructions

GST-importinα1 [49] plasmid was kindly provided by Dr. Yoshihiro Yoneda of Osaka University. Human EGR-1 (residues 338–418), SP1 (residues 626–708) and KLF6 (residues 200–282) were amplified by polymerase chain reactions (PCR) using human cDNA as templates. The sequences of the gene-specific primers are listed in S1 Table. The PCR products were restricted with appropriate enzymes and the fragments were cloned into peGFP-C1 vectors to generate the various GFP-ZFD fusion proteins. Importinβ1 expressed with an N-terminal His-tag (His-Impβ1) was constructed similarly with 2 sets of primers as indicated in S1 Table and inserted into the pCDNA4 vectors. Site-directed mutagenesis was performed by PCR-based QuickChange method (Stratagene), and the primer sequences are listed in S2 Table. All mutations were confirmed by DNA sequencing.

### Subcellular localization analysis

CHO K1 cells (8 x 10^5^) were seeded on coverslip in 24-well dish for 24 h. Transfections were conducted by using Lipofectamine^™^ 2000 with 0.8 μg of plasmid DNA according to the manufacturer's instructions. Cells were transfected for 24 h then rinsed with ice-cold phosphate-buffered saline (PBS) (0.14 M NaCl, 2.7 mM KCl, 1.5 mM KH_2_PO_4_, 8 mM Na_2_HPO_4_, pH 7.4), and fixed with 4% para-formaldehyde in PBS at 4 °C for 30 min. After washing three times with PBS, cells were stained with Hochast 33258 (100 ng/ml) in PBST (PBS with 0.3% Triton^®^ X-100) at room temperature for 15 min. Cells were washed three times with PBS, then mounting medium (90% glycerol, 5% n-propyl gallate) was applied to the coverslips for fluorescence microscopy. The subcellular localization of the GFP-fusion proteins was analyzed with AxioVision Rel. 4.8 (Zeiss). The florescence signals from each cell were quantified as pixel values from the cytoplasm (C) or nucleus (N). GFP-fusion proteins were considered to localize exclusively in nucleus when greater than 90% of the fluorescence signals (C/N pixel values < 10%) came from the nucleus. Results were generated from 30 cells randomly picked from each sample. Statistical analysis was conducted using Student’s *t* test.

### His-tag pull-down assay

CHO K1 cells were co-transfected with GFP-tagged proteins and impβ1-His for 24 h. Cells were lysed and cell lysates were added to microcentrifuge tubes containing 20 μl of cobalt TALON^®^ Metal Affinity resin (Clontech) and 1 ml binding buffer (50 mM NaH_2_PO_4_, 300 mM NaCl, 10 mM imidazole, 0.4% NP-40, 10% glycerol). Tubes were rotated at 4 °C for 2 h. The resins were then washed once with binding buffer, and followed sequentially with binding buffer containing increasing concentrations of imidazole. The wash solutions were collected for immunoblotting.

### GST pull-down assay

GST-impα1 was expressed in *E*. *coli* at 20°C for 14 h by inducing with 1 mM IPTG. The cells were lysed by sonication in extraction buffer (PBS with 0.75 mg/ml lysozyme, 0.5 mM PMSF), and the cell extracts were incubated with 20 μl of Glutathione Sepharose 4B beads and 1 ml binding buffer (20 mM HEPES, 110 mM potassium acetate, 5 mM sodium acetate, 2 mM magnesium acetate, 250 mM sucrose, 0.5 mM EGTA, 2 mM DTT, pH 7.9) for 2 h at 4 °C. The buffer was removed and the beads were further incubated with protein extracts prepared from CHO K1 cells for 2 h at 4 °C. Beads were washed with binding buffer three times, then boiled in SDS buffer and the supernatants were collected for immunoblotting.

### Preparation of whole cell lysates

Cells were harvested by centrifugation at 12,000 x g and resuspended in five volumes of extraction buffer (20 mM HEPES, pH 7.9, 400 mM NaCl, 0.5 mM PMSF, 50 mM NaF, 0.5 mM Na_3_VO_4_, 2 μg/ml aprotinin, 5 μg/ml leupeptin, 1 μg/ml pepstatin, and 0.5% NP-40). Tubes were incubated on ice for 10 min then vortexed vigorously for 5 sec. The vortexing step was repeated four times, and cell debris were removed by centrifugation at 12,000 x g for 20 min. The supernatants were collected for immnoblotting.

### Immunoblotting analysis

Proteins in the supernatants were separated on SDS-polyacrylamide gels (5% stacking and 8% separating gels) then transferred electrophoretically onto PVDF membranes (GE Healthcare) in a transfer cell (Bio-Rad). The membranes were pre-hybridized in TBST buffer (150 mM NaCl, 10 mM Tris, pH 8.0, 0.1% Tween-20) with 5% skim milk for 1 h, and then incubated with primary antibodies in 5% skim milk at 4 °C overnight. After washing three times with TBST buffer, the membranes were submerged in 5% skim milk containing horseradish peroxidase-conjugated secondary antibodies at room temperature for 1 h. After washing three times with TBST buffer, the membranes were rinsed once with TBS buffer (TBST buffer without Tween-20) for 5 min, and developed by the ECL system (Amersham). Visualization was processed by ImageQuantTM LAS 4000 mini imager (GE Healthcare) and the intensities of the bands were quantified with the UN-SCAN-IT gel analysis software (version 6).

### Statistical analysis

Statistical analyses were conducted using the results of three independent experiments. The differences between treatments were determined by using two-tailed Student's *t* test.

## Results

**Fig 1:**
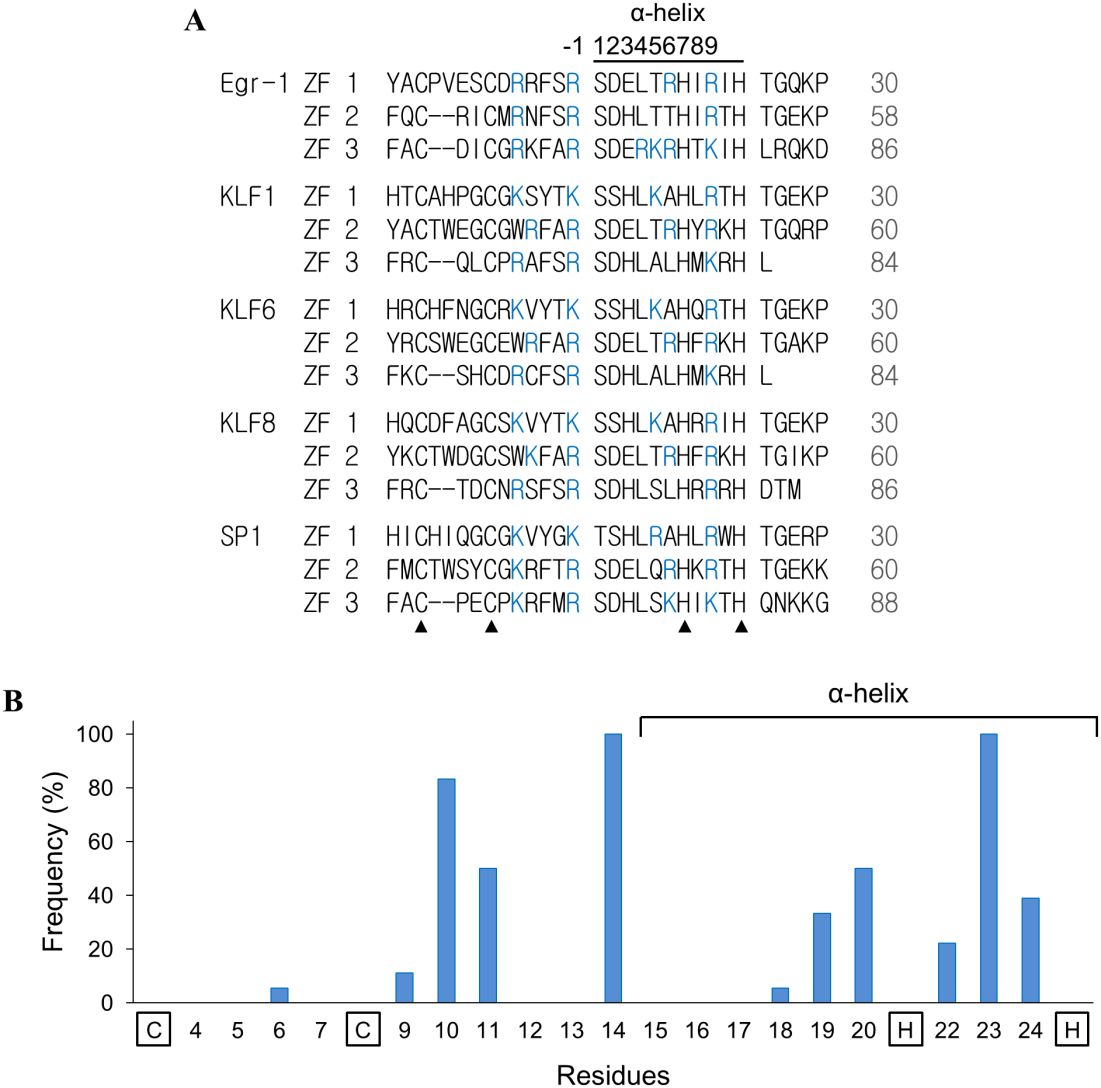
Sequence alignment and analysis of the ZFs from triple C_2_H_2_ ZF proteins with ZFD that reportedly associate with nuclear transport activity. (A) The ZF sequences of human Egr-1 (NP_001955.1), Sp1 (NP_612482.2), KLF1 (NP_006554.1), KLF6 (NP_001291.3), and KLF8 (NP_009181.2) were aligned. Solid triangles indicate the conserved Cys and His residue in the ZFs. (B) The frequency of basic residues appeared in each position of the ZF according to the proteins listed in (A).

Reportedly, ZFD can serve as an NLS and plays a role in nuclear transport. We investigate here the factors that affect the NLS role of the ZFD using ZF proteins with three adjacent C_2_H_2_ ZF motifs. The ZFD sequence of several triple C_2_H_2_ ZF proteins (Egr-1 [38], KLF1 [44], KLF6 [41], KLF8 [50], and SP1 [46]) that reportedly act as NLS were aligned. The residues that modulate the nuclear transport activity are expected to conserve in these ZF motifs. Fig 1A shows the sequence alignments. The residue numbers denoted in Fig 1A refer to the positions of the amino acid residues in the ZFD, not those of the whole proteins. Since basic residues are recognized to involve in nuclear transport, the frequency of a basic amino acid occurs in a specific position is shown (Fig 1B). Noticeably, except the Zn-interacting residues (Cys and His), occurrences of basic residues are rather conserved. The “hot spots” (higher than 80% frequency) at homologous positions of 10, 14 and 23 of each ZF motif (numbered according to the ZF-1 of Erg-1) can be easily detected. Structurally, the α-helical region of the ZF motif encompasses residues 15 to 25 [51], five out of the eight hot and high frequency spots occur in this region.

Despite the high similarity in the distribution of basic residues among ZFs, the contribution of each ZF towards nuclear transport may not be equivalent. We investigated the intrinsic factors that modulate the activity of the ZFs. The ZFD of Egr-1 was used as a model for this study. Egr-1 does not have a classical NLS and thus its ZFD is most likely responsible for its nuclear transport.

**Fig 2:**
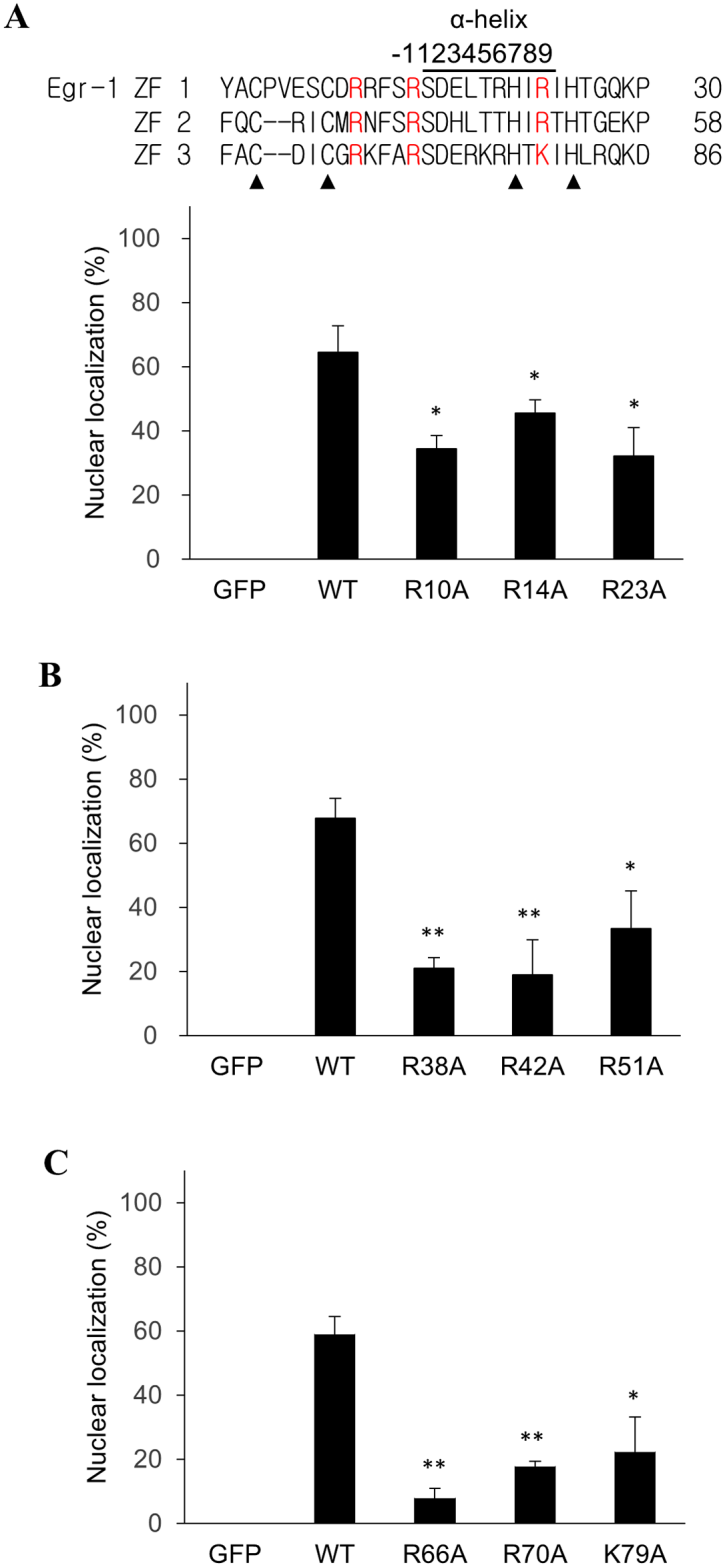
Effect of conserved basic residues in ZFs on the nuclear transport activity of Egr-1 ZFD. Egr-1 ZFD was fused with GFP. The conserved basic residues (hot spots) in ZF1 (A), ZF2 (B) and ZF3 (C) of the fusion proteins were individually mutated to alanine. The plasmids were transfected into CHO K1 cells for protein expression. Egr-1 ZF sequences are shown with the mutated residues in red. Solid triangles indicate the conserved Cys and His residue in the ZFs. The proportion of cells having the GFP fusion protein entirely localized in the nucleus was determined. Representative microscopic images are included as S1 Fig. Each value represents a mean ± standard deviation of three independent experiments. Asterisk indicates significant difference as compared to that of the wild type (WT) fusion protein. *: *p* < 0.05; **: *p* < 0.01.

The sequence of Egr-1 ZFD was amplified from human cDNA and fused with GFP. The construct was used as a template to generate other mutants. To investigate the significance of the “hot spots” in nuclear transport, we replaced individually the three conserved basic residues in every ZF of Egr-1 with alanine. The mutated constructs were transfected and expressed in CHO K1 cells. The proportion of GFP-fusion proteins localize exclusively in the nucleus were recorded. Representative microscopic images in this work are included as S1 Fig. As shown in Fig 2A, GFP by itself does not have nuclear transport activity. The wild type (WT) Egr-1 ZFD fused to GFP presented mainly in the nucleus, indicating the Egr-1 ZFD is responsible for translocating the fusion proteins into the nucleus. Mutation at the hot spots of Egr-1 ZF1 (R10A, R14A and R23A) significantly reduced the nuclear transport activity. Reportedly, ZF1 has limited involvement in the nuclear transport of Egr-1 [38]. Our results indicate that ZF1 does contribute to the nuclear transport activity of Egr-1 ZFD.

The homologous hot spots at the ZF2 and ZF3 of Egr-1 were also mutated. Substitution at any one hot spot of ZF2 (R38A, R42A and R51A) showed a marked reduction in the nuclear transport activity (Fig 2B). The reduction level is more significant than that of ZF1. The same effect was found when similar mutations were generated at ZF3 (R66A, R70A and K79A, Fig 2C). Results from these experiments imply that removing the conserved basic residues on the ZFs can effectively inhibit the nuclear transport activity of the Egr-1 ZFD. In addition, the mutations on ZF2 and ZF3 are more effective than those on ZF1 for this inhibition process.

**Fig 3:**
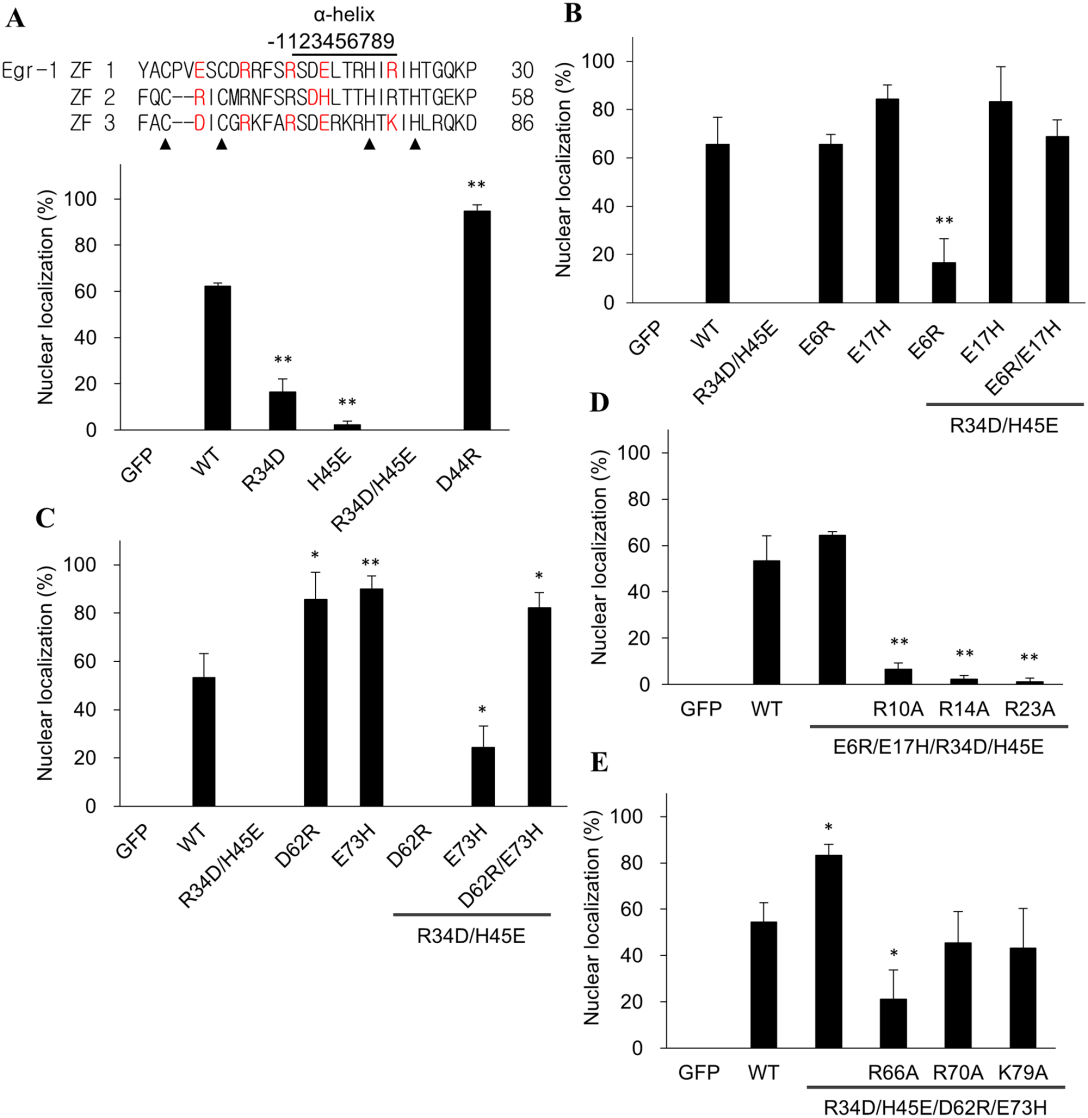
Effect of charge residues in ZFs on the nuclear transport activity of Egr-1 ZFD. Egr-1 ZFD was fused with GFP. (A) Basic (R34 and H45) or acidic (D44) residues in the ZF2 were mutated to oppositely charged residues individually or concurrently. (B) The GFP fusion protein was mutated firstly at ZF2 (R34D/H45E). Additional mutations were performed on ZF1 (E6R, E17H and E6R/E17H). Point mutation (E6R and E17H at ZF1) for the wild type (WT) fusion protein was also conducted. (C) The acidic residue in the ZF3 of the R34D/H45E mutant was further substituted with basic residues (D62R, E73H and D62R/E73H). Point mutation (D62R and E73H at ZF3) for the WT fusion protein was also conducted. (D) The hot spots in the ZF1 or the ZF3 (E) of the E6R/E17H/R34D/H45E mutant protein were further substituted with alanine. Egr-1 ZF sequences are shown on the top panel with the mutated residues in red. Solid triangles indicate the conserved Cys and His residue in the ZFs. The plasmids were transfected into CHO K1 cells for protein expression. The proportion of cells having the GFP fusion protein entirely localized in the nucleus was determined. Representative microscopic images are included as S1 Fig. Each value represents a mean ± standard deviation of three independent samples. Asterisk indicates significant difference as compared to that of the wild type (WT) fusion protein. *: *p* < 0.05; **: *p* < 0.01.

Further sequence analysis reveals that the residues homologous to R34 and H45 of the Egr-1 ZF2 are acidic in ZF1 (E6 and E17) and ZF3 (D62 and E73). R34 is on the linker connecting the 2 β-sheets while H45 is within the α-helix. These acidic residues might interfere with the activity for nuclear entrance. Thus R34 and H45 on ZF2 were replaced with acidic residues individually (R34D and H45E) or concurrently (R34D/H45E). The resulting fusion proteins all have reduced nuclear transport activity. The mutation at H45, which is within the α-helical region, is even more effective than the replacement at the R34 position. Moreover, the effect seems to be synergic since the R34D/H45E double mutant has the lowest nuclear transport activity (Fig 3A). The results imply that basic residues help while acidic residues inhibit the nuclear transport of ZFD proteins. In supporting this hypothesis, the only acidic residue (which occupies the 2^nd^ position on the α-helix) in ZF2 was replaced with arginine (D44R). The resulting fusion protein allocates exclusively in the nucleus (Fig 3A).

As mentioned above, the amino acid residues in ZF1 (E6 and E17) and ZF3 (D62 and E73) homologous to R34 and H45 of ZF2 are acidic and the R34D/H45E double mutant has drastically reduced nuclear transport activity (Fig 3A). We substituted these acidic residues in ZF1 with Arg or His. Fig 3B shows fusion proteins carrying either the E6R or E17H substitution have similar nuclear transport activity as the wild type Egr-1 ZFD. Substitution of E6R, E17H or both replacements to the ZF2 R34D/H45E double mutant partially or completely restored the nuclear transport activity of the fusion proteins. Conversely, replacing the corresponding acidic residues in ZF3 (D62 and E73) with basic residues showed an enhanced nuclear transport activity of the fusion proteins (Fig 3C). Substitution of E73H or both D62R and E73H to the ZF2 R34D/H45E double mutant partially restored or even enhanced the nuclear transport activity of the Egr-1 GFP-ZFD. The results demonstrated that all three ZFs on Egr-1 contribute interactively to the NLS activity of the protein.

The hot spots of ZF1 play a less effectively role in modulating the nuclear transport as compared to that of ZF2 and ZF3 (Fig 2). Since the quadruple mutant (E6R/E17H/R34D/H45E) has restored the nuclear transport activity of the GFP-ZFD fusion protein (Fig 3B), the role of the hot spots in ZF1 of the quadruple mutant was examined. Fig 3D shows that the quadruple mutant carrying additional substitutions at the hot spots of ZF1 has its restored nuclear transport activity diminished. Similar results were found when the hot spots at ZF3 of the quadruple mutant (R34D/H45E/D62R/E73H) were mutated (Fig 3E). Substitution of the hot spot residue to alanine reduced the enhanced nuclear transport activity to a similar or even lower activity than that of the wild type protein. These results show the impact of charge residues in modulating the NLS activity of the ZFD.

**Fig 4:**
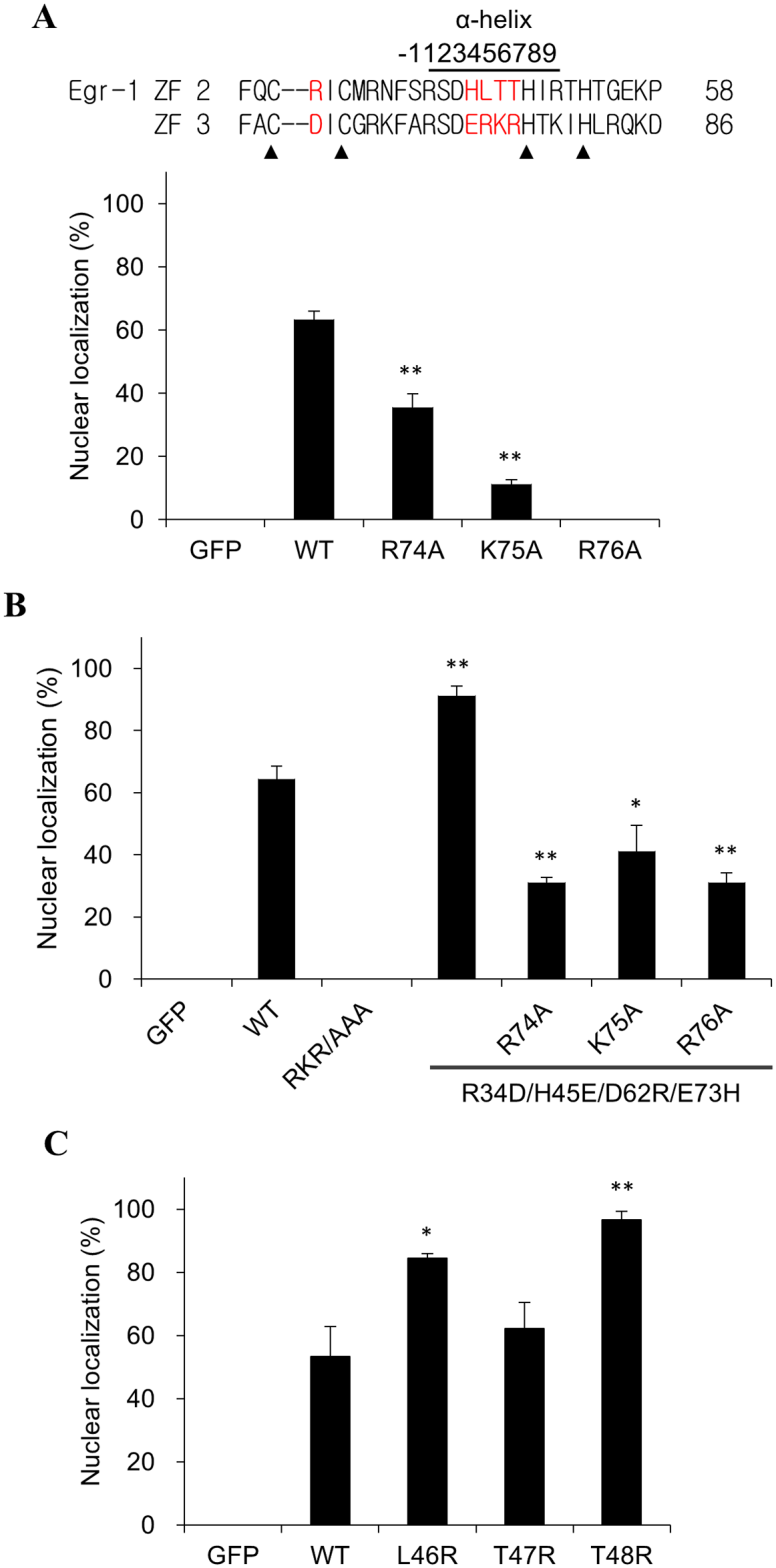
Effect of basic residues at the α-helical region of ZFs on the nuclear transport activity of Egr-1 ZFD. Egr-1 ZFD was fused with GFP. The basic residues at the α4-α6 (^74^RKR^76^) region in the ZF3 of Egr-1 ZFD were substituted with alanine individually (A) or concurrently (RKR/AAA) (B). The hot spot at ZF3 of the R34D/H45E/D62R/E72H mutant protein was mutated to alanine individually. (C) The homologous sequence of ^74^RKR^76^ at ZF2 (46LTT48) in WT Egr-1 ZFD was mutated to arginine individually. Egr-1 ZF sequences are shown on the top panel with the mutated residues in red. Solid triangles indicate the conserved Cys and His residue in the ZFs. The plasmids were transfected into CHO K1 cells for protein expression. The proportion of cells having the GFP fusion protein entirely localized in the nucleus was determined. Representative microscopic images are listed as S1 Fig. Each value represents a mean ± standard deviation of three independent samples. Asterisk indicates significant difference as compared to that of the wild type (WT) fusion protein. *: p < 0.05; **: p < 0.01.

The α-helical region of the ZF interacts with the major groove of target DNA. Three basic residues in tandem (^74^RKR^76^, α4-α6 of the helix) can be found in the ZF3 of Egr-1. Mutation of these three residues to alanine individually resulted in a dramatic reduction of the nuclear transport activity (Fig 4A). The effect was most prominent with the mutation at α6. Since quadruple mutant (R34D/H45E/D62R/E73H) has an enhanced nuclear transport activity (Fig 3C, 6^th^ column), and E73 is located right next to the ^74^RKR^76^ sequence, the role of ^74^RKR^76^ was examined individually. Mutation at any of the basic residue significantly reduced the activity of the quadruple (R34D/H45E/D62R/E73H) mutant. However, the reduction was not as significant as that of wild type (Fig 4B). The protein levels of the wild type fusion proteins and mutants in the cells are similar. The reduction in transport efficiency is not due to the lack of fusion proteins carrying multiple mutations.

The homologous sequence of ^74^RKR^76^ at ZF2 is ^46^LTT^48^ and does not carry any positively charged side chains. The L46R and T48R mutation enhanced the nuclear localization activity of the Egr-1 ZFD (Fig 4C). Apparently, a positive charge at the 4^th^ to 6^th^ position of the α-helix on ZF2 can improve the nuclear transport activity of Erg-1. The results indicate the impact of basic residues at hot spots and α-helix for the nuclear transport activity of ZFs. Whether this mutation affects the DNA binding activity or specificity of Erg-1 remains to be investigated.

**Fig 5:**
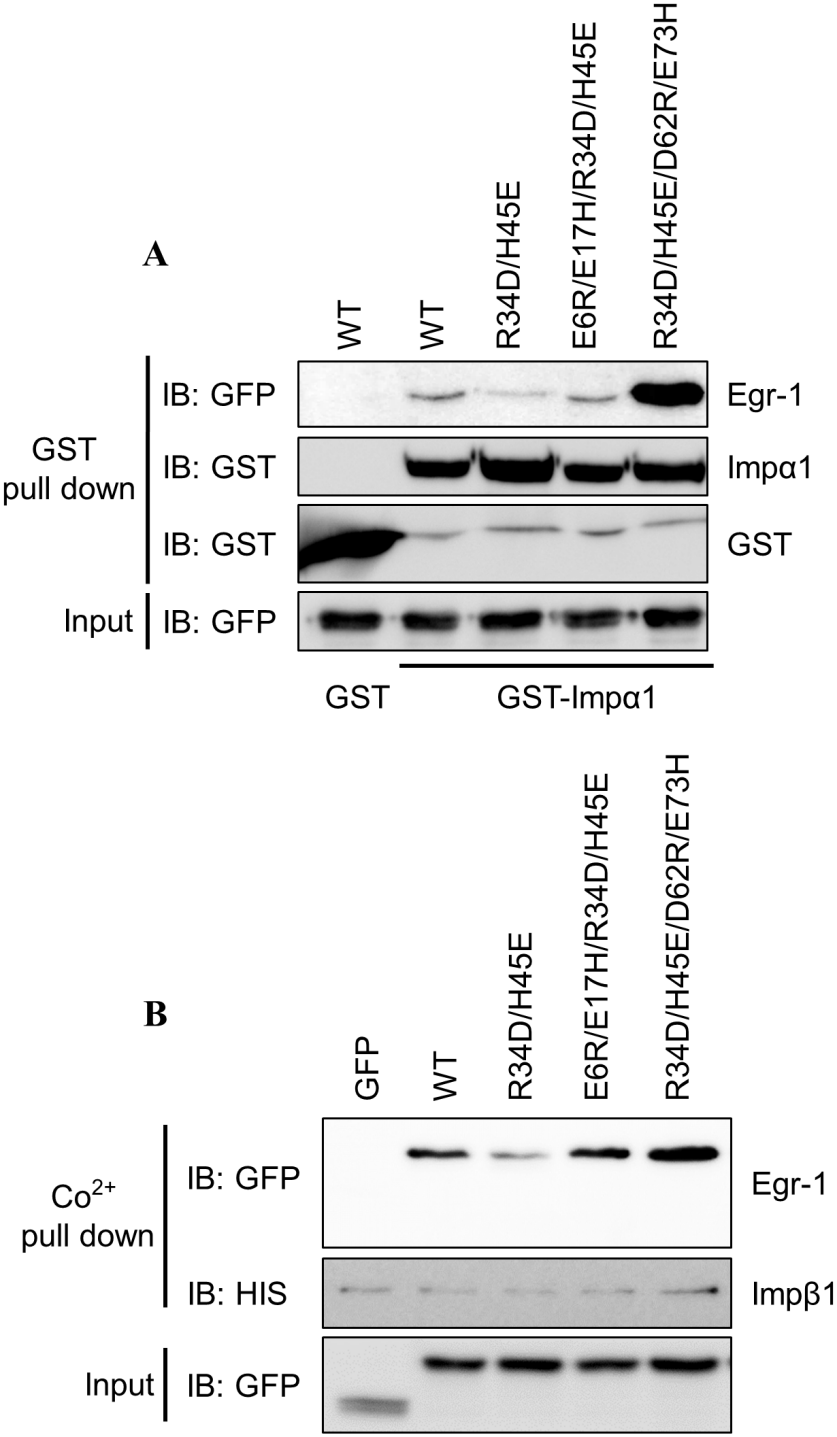
The binding affinity of nuclear transport proteins with Egr-1 GFP and the mutated proteins. (A) Cell extracts containing GFP-Egr-1 ZFD fusion protein or its mutants were incubated with extracts expressing GST-Impα1. The interactions between the fusion proteins and GST-Impα1 were analyzed by the GST pull-down assay. (B) GFP-Egr-1 ZFD fusion protein or its mutants were co-expressed with His-tagged Impβ1. The interactions between the fusion proteins and Impβ1 were analyzed by the His-tag pull-down assay.

Entrance of proteins through nuclear pore complex requires the assistance of importins. Reportedly, several C_2_H_2_ ZF proteins interact with importins for nuclear translocation [43, 47, 48, 50]. Since the charge residues in the Egr-1 ZFD affect the NLS activity, we examined whether the effect is derived from the interaction of ZFs with importins. A GST pulldown assay was utilized to examine the interactions. GST-fused Importinα1 (GST-Impα1) was expressed in *E*. *coli*, and GFP-fused Egr-1 ZFD was synthesized in CHO K1 cells. Cell extracts were prepared and mixed to conduct the assay. As shown in Fig 5A, Egr-1 ZFD can interact with Impα1. The interaction reduced significantly for the R34D/H45E mutant, indicating the presence of acidic residues at these positions affect the interaction. However, this interaction was restored for the E6R/E17H/R34D/H45E and the R34D/H45E/D62R/E73H mutants. A higher binding capacity was noted for R34D/H45E/D62R/E73H which corresponds to the higher nuclear transport activity for this mutated protein (Fig 3E, 3^rd^ column). These results suggest the contribution of the charged residues on ZFs in nuclear transport.

Since Impα1 usually interacts with Impβ in the nuclear transport process. The interaction between ZFD and Impβ1 was also analyzed. Impβ1 was fused with 6 histidine residues (Impβ1-His) and co-expressed with the GFP-fused Egr-1 ZFD constructs in CHO K1 cells. The interaction can be revealed by cobalt beads pull-down assay. Fig 5B shows the interaction of Egr-1 ZFD and Impβ1. A similar result as that of Fig 5A was obtained. The R34D/H45E mutant, but not the E6R/E17H/R34D/H45E and the R34D/H45E/D62R/E73E mutants, showed a decrease of interaction with Impβ1 as compared to that of wild type Egr-1 ZFD. The result implies that both importins are required for the nuclear transport of the Egr-1 ZFD.

**Fig 6:**
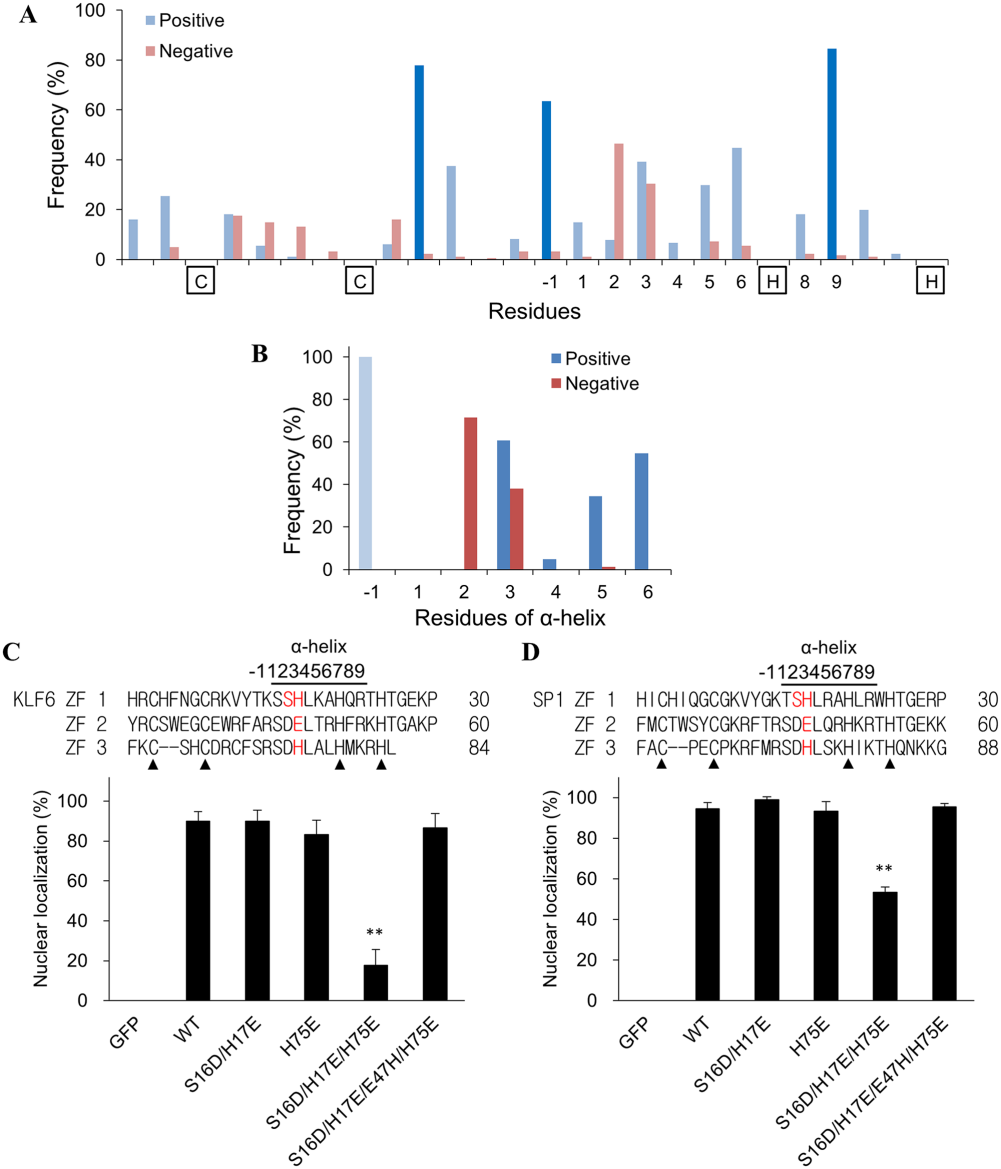
Analysis of acidic residues in the α-helical region of triple C_2_H_2_ ZFD and their role in nuclear transport. (A) The ZF sequences of 61 triple C_2_H_2_ ZF proteins (183 ZFs) were retrieved from the Uniprot database. The frequency of charge residue distribution in each position was analyzed. The basic and acidic residues are highlighted in blue and red colors, respectively. (B) The sequences of the α-helical region of the Eger/Sp1/KLF family were analyzed. The frequencies of occurrence of basic and acidic residues at each position are represented in blue and red colors, respectively. GFP was fused with KLF6 (C) or SP1 (D) ZFD. The α2 and/or α3 of the α-helix of KLF6 or SP1 ZFD were mutated to acidic residues. The construct with acidic residues (DE at α2-α3) for all three ZFs (S16D/H17E/H75E) was further mutated at E47 (S16D/H17E/E47H/H75E). Egr-1 ZF sequences were shown on the top panel with the mutated residues in red. Solid triangles indicate the conserved Cys and His residue in the ZFs. The sequence of the KLF6 or SP1 ZFD is shown in the upper panel of each figure with the mutated residues in red. Solid triangles indicate the conserved Cys and His residue in the ZFs. The plasmids were transfected into CHO K1 cells for protein expression. The proportion of cells having the GFP fusion protein entirely localized in the nucleus was determined. Representative microscopic images are listed as S1 Fig. Each value represents a mean ± standard deviation of three independent samples. Asterisk indicates significant difference as compared to that of the wild type (WT) fusion protein. **: *p* < 0.01.

The above results show that charge residues in the ZFD of triple C_2_H_2_ ZF proteins are critically associated with nuclear transport. We examined the distribution and proportion of charge residues in the ZFs. Sixty-one triple C_2_H_2_ ZF proteins (183 ZF motifs) were identified and retrieved from the Uniprot database [52]. The hot spots are conserved in these ZF proteins (Fig 6A), indicating the significance of the hot spots in nuclear transport. Besides, a marked proportion of charge residue locates at the α-helix of the ZFs. Acidic residues are mainly at the α2 and α3, while the basic residues are found at the α3, α5 and α6 positions. Noticeably, the basic residue at α3 is exclusively His while Arg and Lys residues are mainly at α5 and α6. This finding indicates that the charge residues in the hot spots and the α-helix of ZF motif are important for the NLS function.

Further analysis of the acidic residue in the α-helix reveals that 96% (81/84) of α2 and 85% (46/55) of α3 residue is Asp and Glu, respectively. These DE residues (α2-α3) present in the ZF1 and ZF3 of Egr-1 ZFD (Fig 1). Mutation at the α3 position of ZF2 (H45E) greatly reduced the NLS activity (Fig 3A). On the contrary, replacing the α2 with basic residue (D44R) significantly enhanced the nuclear transport activity of the Egr-1 ZFD (Fig 3A). We examined the occurrence of DE at α2-α3 of the triple C_2_H_2_ ZF proteins and found that this combination can only be found in the ZFs of Egr, SP1 and KLF protein families. Further analysis of residues in the α-helix of those ZFs showed a similar pattern as that depicts in Fig 6A (Fig 6B). Noticeably, the α2-α3 combination is consistently SH (ZF1), DE (ZF2) and DH (ZF3) for SP1 and KLF families. Although a high proportion of His residue presents at α3, changing SH to DE (S16D/H17E) or DH to DE (H75E) in KLF6 did not alter the nuclear transport activity (Fig 6C). Concurrent substitution of the α2-α3 to DE (S16D/H17E/H75E) resulted in a marked reduction of the nuclear transport activity. However, further substitution of DE at ZF2 of KLF6 to DH (S16D/H17E/E47H/H75E), the same α2-α3 charge distribution as that of Egr-1 ZFD, restored the activity (Fig 6C).

Experiments utilizing SP1 ZFD to conduct the experiment generate similar results; the nuclear transport activity dropped only when the DE sequence presents in the α2-α3 of every ZF (Fig 6D). The activity reduced further when α6 (K78) of SP1 ZF3 was mutated (S2 Fig). Applying GST pull down assay to examine the interaction of the KLF6/SP1 ZFD with Impα1 reveals that the interaction reduced with the DE residues presented at the α2-α3 of every ZF (S3 Fig). The interaction restored when the DE at ZF2 was further substituted with DH. Besides the acidic residues, substitution of basic residue (R50A) at α6 of the S16D/H17E/E47H/H75E mutant decreased the restored NLS activity again for KLF6 or SP1 ZFDs (Panels A and B in S4 Fig). These results indicate the influence of charge residues at the α-helix of ZF in regulating the nuclear transport activity.

## Discussion

Accumulated studies indicate that ZFD can be an NLS to facilitate nuclear translocation. However, the transport mechanism remains unclear. The configurations of the C_2_H_2_ ZFs are similar and conserved amino acid residues can be identified among their sequences. Results from this study show that charged residues in the triple C_2_H_2_ ZFD are associated with nuclear transport activity. Although the distribution of basic residues is conserved in the ZFs (Figs 1A and 6A), not every ZF contributes equally to the NLS activity. We showed in this study that the conserved basic residues (hot spots) and the charge residues in the α-helical region modulate the NLS activity of the triple C_2_H_2_ ZF proteins.

Distribution of the charge residues at the α-helical region varies among the C_2_H_2_ ZFs. For the Egr/SP1/KLF families, the charge residues present within the second (α2) to the sixth (α6) position of the α-helix affects the NLS activity of the ZFs. The acidic residue at the α2 and α3 positions may reduce the activity while the basic residue at α5 and α6 enhance nuclear entry. The α-helix constitutes a small region but exposed on the surface of the ZF. Since we showed clearly that the ZFs interact with importins and the interactions determine the activity of nuclear entry, the locations of the charge residues should be at sites that can be in contact with the carrier proteins.

The classical NLS has a cluster of basic residues which is the major feature recognized by the transporter proteins. ZFs do not have this recognition motif. However, the ZFD can sterically align the basic residues that are distributed at different ZFs to form a pattern to be recognized and interacted with the transporter proteins. Probably, this is the reason why a single C_2_H_2_ ZF is not sufficient to support the activity of nuclear entry. It should be noted that not every ZF in the multiple C_2_H_2_ ZFs is required to participate in the NLS activity. A variety of studies indicate that only 3 to 4 consecutive ZFs in a multiple C_2_H_2_ ZF proteins are involved in the NLS activity [35, 36, 53]. Since the classical NLS is composed of 4–5 basic residues, a few adjacent ZFs are sufficient to generate a monopartite- or bipartite-like construct for nuclear transport.

Results from our study also reveal that the NLS role of ZF is interchangeable. Egr-1 ZF2 plays a significant role in NLS activity. Mutation at ZF2 abolished the nuclear entry (Fig 3A). However, changing the characteristics of charge residue distribution in ZF1 to mimic that of ZF2 (E6R/E17H/R34D/H45E) restored the NLS activity (Fig 3B). The “functional” ZFs for nuclear transport switched from ZF2 to ZF1. For SP1/KLF family, at least one basic residue is required to present at the α3 position of the ZF to gain the full NLS activity; this basic residue can be in any of the three ZFs (Fig 6). Since changes in Egr-1 and SP1/KLF6 can alter the importin binding ability (Fig 5 and S2 Fig), the results indicate that the contact sites between ZFD and importin are interchangeable. The importin accesses the ZFD and interacts with the NLS region. Mutation at the NLS interface can either alters the interaction or generates a new site for the interaction. Alternatively, importin recognizes a broad region of the ZFD and a limited number of interactions are sufficient to form and stabilize the protein complex for nuclear transport.

ZFD is recognized initially as a DNA binding domain. Since accumulated results demonstrate that ZFD can serve as an NLS for nuclear transport, some residues in the triple C_2_H_2_ ZFD may have dual functions. The interaction of Egr-1 or SP1 ZFD with the DNA of target promoter has been explored [54, 55]. Examining the protein-DNA contact sites reveals that the first and the second hot spot of every ZF are involved in DNA binding. In addition, DE residues in the α-helix (α2-α3) of ZF1 are required for Egr-1 to bind target promoter [56]. The DE residues in the ZF2 of SP1 recognize specific DNA in the target promoter [54]. Results from these studies indicate the significance and the dual roles of the charge residues in nuclear transport and DNA binding activities.

The three-dimensional (3D) structure of a triple C_2_H_2_ ZFD and importin interaction has not been reported. However, the crystal structure of Snail1 ZFD complexed with Impβ1 has been solved [48]. Snail1 has 4 ZFs and the ZFD provides the NLS activity. Each of the ZF in Snail1 is in contact with Impβ1 and thus participates in nuclear transport. All of the amino acid residues involved in the NLS activity locates at the hot spots and the α-helical region of each ZF. These residues aligned to form an interface as revealed by the 3D structure. The hot spots at the ZFs of Snail1 are conserved with those of triple C_2_H_2_ ZFD. Noticeably, the second and the third hot spot of ZF2, the first and the third hot spot of ZF3 and the second hot spot of ZF4 are part of the NLS and interact with the acidic residues on Impβ1. Other contact sites include α1 to α6 of the helices although not every residue is basic [48]. Even though the configuration and substrate interacting sites between Snail1 and triple C_2_H_2_ ZFD are different, the structural analysis of Snail1 implicates the critical role of hot spots and residues in the α-helical region of the protein in the nuclear transport of ZFs. It is reasonable to expect that residues involved in the nuclear transport of the triple C_2_H_2_ ZFs aligned at the same interface and they are accessible to the transport proteins. Since our results also indicate that the contributions among ZFs in nuclear transport can be interchanged, the interacting sites between ZFs and importins can vary. Certainly, we can exclude the possibility that there are multiple recognition sites between ZFs and importins and not all the recognition sites have to be interacted with importins to form a stable complex for nuclear transportation.

The SP1 and KLF6 have a ZFD with strong nuclear entry activity (Fig 6C and 6D). Both proteins have an acidic residue at the α3 of ZF2 but a basic residue at both ZF1 and ZF3. Mutation at the α3 of either the ZF1 or ZF3 to acidic residue did not alter the NLS activity. Egr-1 only has a basic residue at the α3 (H45) among the ZFs. Substitution of the amino acid with an acidic residue (H45E) drastically reduced nuclear transport activity (Fig 3A). Switching the basic residue at ZF2 with the acidic residue at ZF1 did not affect the NLS activity (Fig 3B). Possibly, at least one basic residue at the α3 of any ZF is sufficient to maintain the NLS activity of the ZFD.

Recently, C_2_H_2_ ZFD is broadly used in the application of cell engineering [57, 58]. Besides capable of specific DNA-binding, ZFD has cell penetration activity [59]. Study showed that classical NLS can serve as a cell penetration peptide via the action of basic amino acid residues [60]. The positively charged residues in ZFD might also play a similar role [61]. As discussed above, the hot spot and basic residues in the α-helical region of ZFs are involved in nuclear transport and DNA-binding activities. These residues have been suggested to associate with cell penetration [61]. Therefore, the basic residues on ZFD may have overlapping functions in cell permeability, nuclear transport and DNA binding. These findings can provide information for the design of functional tools in cell engineering.

In summary, we show herein the critical factors modulating the NLS activity of triple C_2_H_2_ ZF proteins. The conserved basic residues and the charge residues at the α-helical region of the ZFs regulate the nuclear transport activity. Presently, the conclusion is drawn from the results derived from triple C_2_H_2_ ZF proteins, especially on the Egr/SP1/KLF families. Mutations at the conserved hot spots and the basic residues in the α-helix reduce the nuclear transport activity. Whether this conclusion can be applied to other multiple ZF proteins remains to be investigated.

## Supporting information

S1 Table Primer sequences for fusion protein construction. (DOCX)

S2 Table Primer sequences used in ZFD mutagenesis. (DOCX)

S1 Fig Representative microscopic images in this study. The nuclei were stained with DAPI (blue). Scale bars, 10 μm.
(PDF)

S2 Fig Effect of Lys78 in the α-helical region of ZF3 on the nuclear transport activity of SP1 ZFD. The Lys78 (α6) in the α-helical region of SP1 were mutated to alanine with S16D/H17E or S16D/H17E/H75E. The plasmids were transfected into CHO K1 cells for protein expression. The proportion of cells having the GFP fusion protein entirely localized in the nucleus was determined. Each value represents a mean ± standard deviation of three independent samples. Asterisk indicates significant difference as compared to that of the wild type (WT) fusion protein. *: p < 0.05; **: p < 0.01. SP1 ZF sequences are shown on the top panel with the mutated residues in red. Solid triangles indicate the conserved Cys and His residue in the ZFs. Representative microscopic images are shown in the middle panel. Scale bars, 10 μm.
(TIF)

S3 Fig The binding affinity of Impα1 with KLF6 and SP1. Cell extracts containing wild type and mutated GFP-KLF6 ZFD (A) or GFP-SP1 ZFD (B) fusion protein were incubated with extracts expressing GST-Impα1. The interactions between the fusion proteins and GST-Impα1 were analyzed by the GST pull-down assay.
(TIF)

S4 Fig Effect of charge residue in the α-helical region of ZF on the nuclear transport activity of KLF6 and SP1 ZFD. The α2-α3 residues in the α-helical region of KLF6 (A) or SP1 (B) were mutated (S16D/H17E/E47H/H75E) to mimic that of Egr-1 (DE, DH and DE at α2-α3 of ZF1, ZF2 and ZF3, respectively). The basic residue at α6 of the ZF2 was further mutated (R50A). The plasmids were transfected into CHO K1 cells for protein expression. The proportion of cells having the GFP fusion protein entirely localized in the nucleus was determined. Each value represents a mean ± standard deviation of three independent samples. Asterisk indicates significant difference as compared to that of the wild type (WT) fusion protein. **: *p* < 0.01. The sequence of the KLF6 or SP1 ZFD is shown in the upper panel of each figure with the mutated residues in red. Solid triangles indicate the conserved Cys and His residue in the ZFs. Representative microscopic images are shown in the left panel of each figure. Scale bars, 10 μm.
(TIF)
