# Supplementary material for: The conserved basic residues and the charged amino acid residues at the α-helix of the zinc finger motif regulate the nuclear transport activity of triple C2H2 zinc finger proteins
Source: PLoS One. 2018 Jan 30;13(1):e0191971. doi: 10.1371/journal.pone.0191971 (PMC5790263; doi:10.1371/journal.pone.0191971)
Supplement: S1 Table — (DOCX) [file pone.0191971.s001.docx]

| S1 Table. Primer sequences for fusion protein construction. | | | |
| --- | --- | --- | --- |
| Name | Enzyme site |  | Sequence (5'-3') |
| Egr-1 | BglII | F | ACTCAGATCTTACGCTTGCCCAGTGGAGTC |
|  | HindIII | R | TTCGAAGCTTGTCCTTCTGCCGCAAGTGGA |
| SP1 | BglII | F | ACTCAGATCTCATATTTGCCACATCCAAGG |
|  | EcoRI | R | TGCAGAATTCTCCCTTCTTATTCTGGTGGG |
| KLF6 | BglII | F | ACTCAGATCTCACCGGTGCCACTTTAACGG |
|  | EcoRI | R | TGCAGAATTCGAGGTGCCTCTTCATGTGCA |
| Imp beta1 | KpnI | F | GCTTGGTACCATGGAGCTGATCACCATTCT |
| Imp beta1 middle | KpnI | R | AGCCATCACTGCTGCATCCCGGTACCGCCA |
| Imp beta1 middle | KpnI | F | TGGCGGTACCGGGATGCAGCAGTGATGGCT |
| Imp beta1 | AgeI | R | GATGACCGGTAGCTTGGTTCTTCAGTTTCC |
|  | | | |
