# Supplementary material for: The conserved basic residues and the charged amino acid residues at the α-helix of the zinc finger motif regulate the nuclear transport activity of triple C2H2 zinc finger proteins
Source: PLoS One. 2018 Jan 30;13(1):e0191971. doi: 10.1371/journal.pone.0191971 (PMC5790263; doi:10.1371/journal.pone.0191971)
Supplement: S2 Table — (DOCX) [file pone.0191971.s002.docx]

| S2 Table. Primer sequences used in ZFD mutagenesis. | | | | |
| --- | --- | --- | --- | --- |
| Name | Site mutagenesis | |  | Sequence (5'-3') |
| Egr-1 | ZF1 | E6R | F | CTTGCCCAGTGAGGTCCTGTGATCG |
|  |  |  | R | CGATCACAGGACCTCACTGGGCAAG |
|  |  | R10A | F | GAGTCCTGTGATGCCCGCTTCTCCC |
|  |  |  | R | GGGAGAAGCGGGCATCACAGGACTC |
|  |  | R14A | F | CGCCGCTTCTCCGCCTCCGACGAGC |
|  |  |  | R | GCTCGTCGGAGGCGGAGAAGCGGCG |
|  |  | R14A | F | CGCCGCTTCTCCGCCTCCGACCACC |
|  |  | (with E17H) | R | GGTGGTCGGAGGCGGAGAAGCGGCG |
|  |  | E17H | F | CTTCTCCCGCTCCGACCACCTCACCCGCCACATC |
|  |  |  | R | GATGTGGCGGGTGAGGTGGTCGGAGCGGGAGAAG |
|  |  | R23A | F | CACCCGCCACATCGCCATCCACACAGG |
|  |  |  | R | CCTGTGTGGATGGCGATGTGGCGGGTG |
|  | ZF2 | R38A | F | CGCATCTGCATGGCCAACTTCAGCC |
|  |  |  | R | GGCTGAAGTTGGCCATGCAGATGCG |
|  |  | R42A | F | CGCAACTTCAGCGCCAGCGACCACCTC |
|  |  |  | R | GAGGTGGTCGCTGGCGCTGAAGTTGCG |
|  |  | D44R | F | CTTCAGCCGCAGCAGACACCTCACCACCC |
|  |  |  | R | GGGTGGTGAGGTGTCTGCTGCGGCTGAAG |
|  |  | L46R | F | CAGCGACCACCGAACCACCCACATC |
|  |  |  | R | GATGTGGGTGGTTCGGTGGTCGCTG |
|  |  | T47R | F | CGACCACCTCAGAACCCACATCCGC |
|  |  |  | R | GCGGATGTGGGTTCTGAGGTGGTCG |
|  |  | T48R | F | CCACCTCACCAGACACATCCGCACC |
|  |  |  | R | GGTGCGGATGTGTCTGGTGAGGTGG |
|  |  | R51A | F | CACCTCACCACCCACATCGCAACCCACACAGGCGAAAAG |
|  |  |  | R | CTTTTCGCCTGTGTGGGTTGCGATGTGGGTGGTGAGGTG |
|  | ZF3 | D62R | F | GAAAAGCCCTTCGCCTGCAGAATCTGTGGAAGAAAGTTTG |
|  |  |  | R | CAAACTTTCTTCCACAGATTCTGCAGGCGAAGGGCTTTTC |
|  |  | R66A | F | CCTGCGACATCTGTGGAGCAAAGTTTGCCAGGAGCG |
|  |  |  | R | CGCTCCTGGCAAACTTTGCTCCACAGATGTCGCAGG |
|  |  | R66A | F | CTGCAGAATCTGTGGAGCCAAGTTTGCCAGGAGCG |
|  |  | (with D62R) | R | CGCTCCTGGCAAACTTGGCTCCACAGATTCTGCAG |
|  |  | R70A | F | GGAAGAAAGTTTGCCGCGAGCGATGAACGCAAG |
|  |  |  | R | CTTGCGTTCATCGCTCGCGGCAAACTTTCTTCC |
|  |  | R70A | F | GAAGAAAGTTTGCCGCGAGCGATCACCGC |
|  |  | (with E73H) | R | GCGGTGATCGCTCGCGGCAAACTTTCTTC |
|  |  | E73H | F | GTTTGCCAGGAGCGATCACCGCAAGAGGCATACC |
|  |  |  | R | GGTATGCCTCTTGCGGTGATCGCTCCTGGCAAAC |
|  |  | R74A | F | CCAGGAGCGATGAAGCCAAGAGGCATACC |
|  |  |  | R | GGTATGCCTCTTGGCTTCATCGCTCCTGG |
|  |  | R74A | F | CCAGGAGCGATCACGCGAAGAGGCATACCAAG |
|  |  | (with E73H) | R | CTTGGTATGCCTCTTCGCGTGATCGCTCCTGG |
|  |  | K75A | F | GGAGCGATGAACGCGCCAGGCATACCAAGATC |
|  |  |  | R | GATCTTGGTATGCCTGGCGCGTTCATCGCTCC |
|  |  | K75A | F | GGAGCGATCACCGCGCAAGGCATACCAAGATC |
|  |  | (with E73H) | R | GATCTTGGTATGCCTTGCGCGGTGATCGCTCC |
|  |  | R76A | F | GCGATGAACGCAAGGCGCATACCAAGATCC |
|  |  |  | R | GGATCTTGGTATGCGCCTTGCGTTCATCGC |
|  |  | R76A | F | CGATCACCGCAAGGCGCATACCAAGATCC |
|  |  | (with E73H) | R | GGATCTTGGTATGCGCCTTGCGGTGATCG |
|  |  | RKR/AAA | F | CCAGGAGCGATCACGCTGCCGCGCATACCAAGATCC |
|  |  |  | R | GGATCTTGGTATGCGCGGCAGCGTGATCGCTCCTGG |
|  |  | K79A | F | GCAAGAGGCATACCGCAATCCACTTGCGGCAG |
|  |  |  | R | CTGCCGCAAGTGGATTGCGGTATGCCTCTTGC |
| KLF6 | ZF1 | S16D/H17E | F | GTTTACACCAAAAGCGACGAATTGAAAGCACACCAG |
|  |  |  | R | CTGGTGTGCTTTCAATTCGTCGCTTTTGGTGTAAAC |
|  | ZF2 | E47H | F | GCAAGAAGTGATCACTTAACCAGGCAC |
|  |  |  | R | GTGCCTGGTTAAGTGATCACTTCTTGC |
|  |  | R50A | F | GAAGTGATGAGTTAACCGCGCACTTCCGAAAGCAC |
|  |  |  | R | GTGCTTTCGGAAGTGCGCGGTTAACTCATCACTTC |
|  |  | R50A | F | GAAGTGATCACTTAACCGCGCACTTCCGAAAGCAC |
|  |  | (with ZF2DH) | R | GTGCTTTCGGAAGTGCGCGGTTAAGTGATCACTTC |
|  | ZF3 | H75E | F | GTTTTTCCAGGTCTGACGAACTGGCCCTGCACATGAAG |
|  |  |  | R | CTTCATGTGCAGGGCCAGTTCGTCAGACCTGGAAAAAC |
| SP1 | ZF1 | S16D/H17E | F | GAAAGTGTATGGCAAGACCGACGAACTGCGGGCACACTTGCGC |
|  |  |  | R | GCGCAAGTGTGCCCGCAGTTCGTCGGTCTTGCCATACACTTTC |
|  | ZF2 | E47H | F | CGCTTCATGAGGAGTGACGAGCTGTCAAAACATATCAAG |
|  |  |  | R | CTTGATATGTTTTGACAGCTCGTCACTCCTCATGAAGCG |
|  |  | R50A | F | CGGATGAGCTACAGGCGCACAAACGTACAC |
|  |  |  | R | GTGTACGTTTGTGCGCCTGTAGCTCATCCG |
|  |  | R50A | F | CGGATCACCTACAGGCGCACAAACGTACAC |
|  |  | (with E47H) | R | GTGTACGTTTGTGCGCCTGTAGGTGATCCG |
|  | ZF3 | H75E | F | CTTCACACGTTCGGATCACCTACAGAGGCACAAACG |
|  |  |  | R | CGTTTGTGCCTCTGTAGGTGATCCGAACGTGTGAAG |
|  | | | | |
