# Supplementary material for: The conserved basic residues and the charged amino acid residues at the α-helix of the zinc finger motif regulate the nuclear transport activity of triple C2H2 zinc finger proteins
Source: PLoS One. 2018 Jan 30;13(1):e0191971. doi: 10.1371/journal.pone.0191971 (PMC5790263; doi:10.1371/journal.pone.0191971)

Microscopy images for Fig 2A

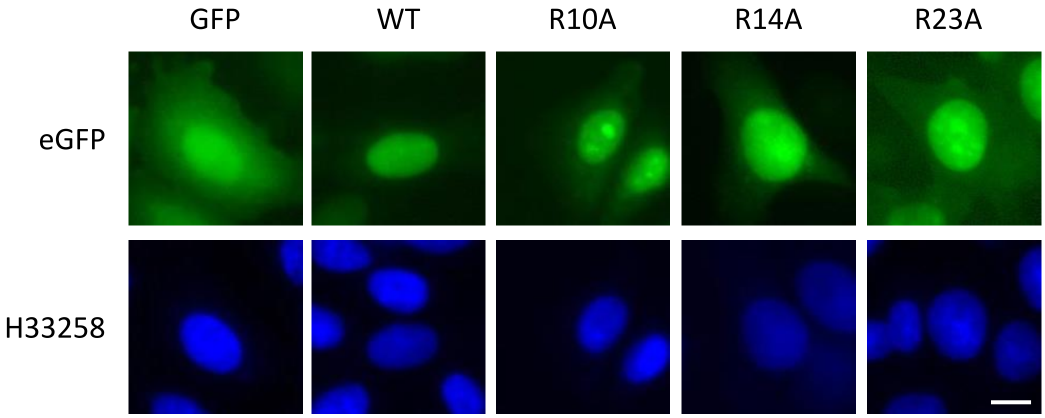

Microscopy images for Fig 2B

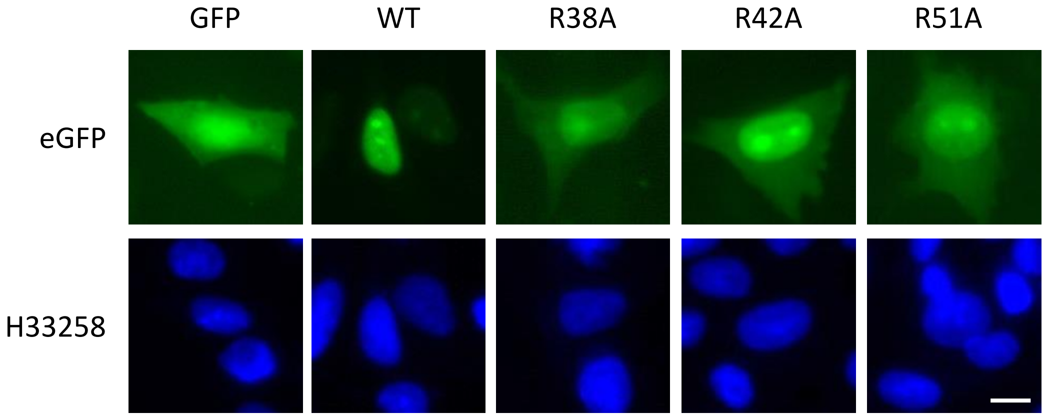

Microscopy images for Fig 2C

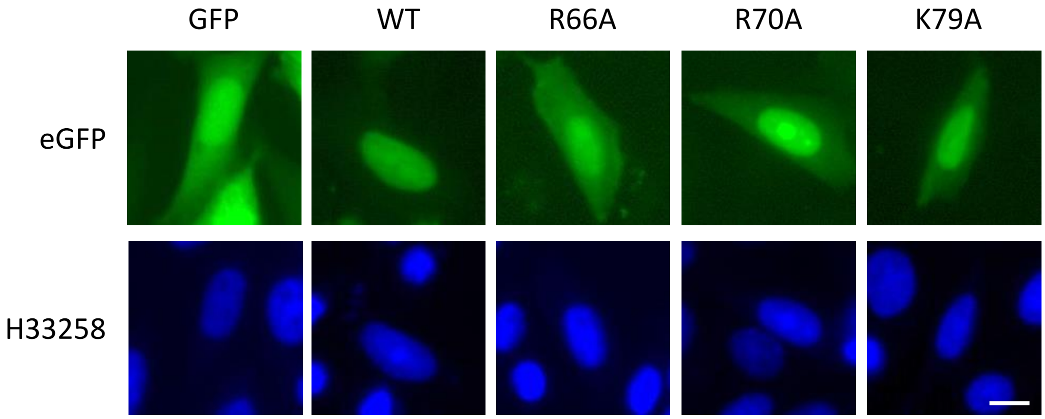

Microscopy images for Fig 3A

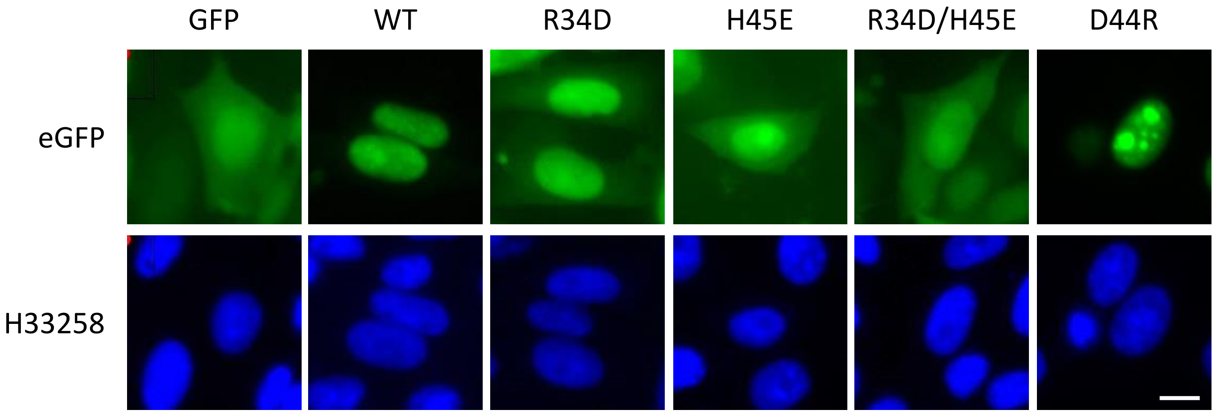

Microscopy images for Fig 3B

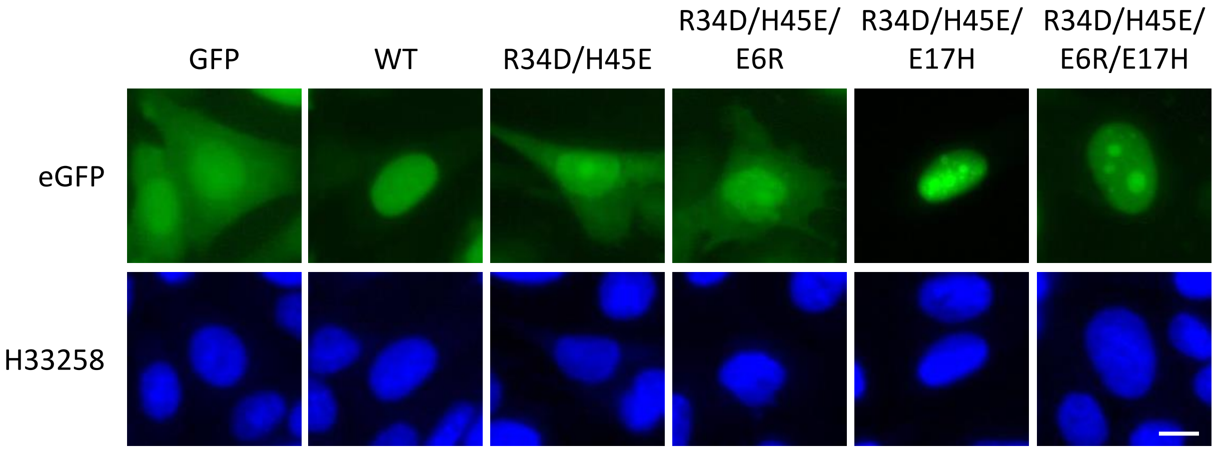

Microscopy images for Fig 3C

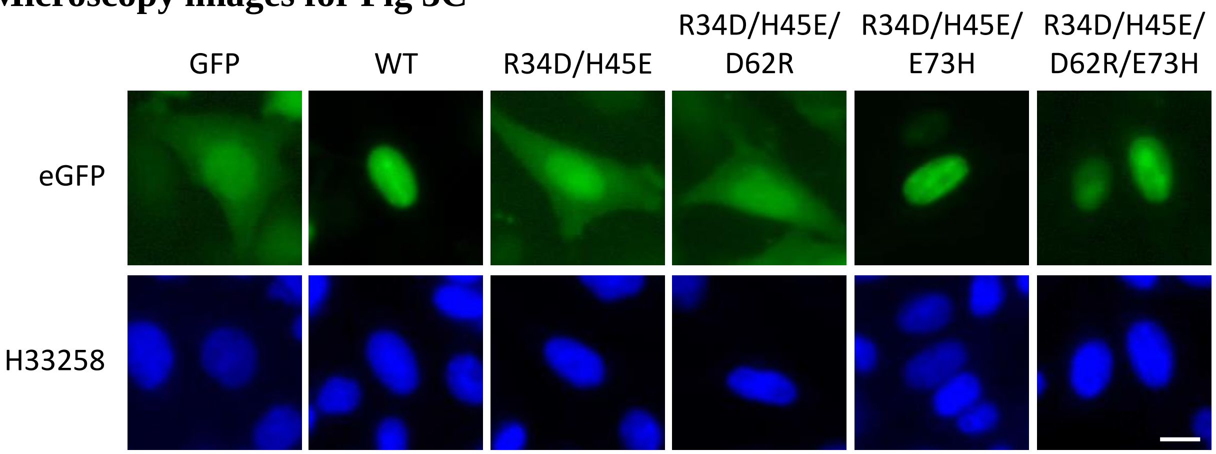

Microscopy images for Fig 3D

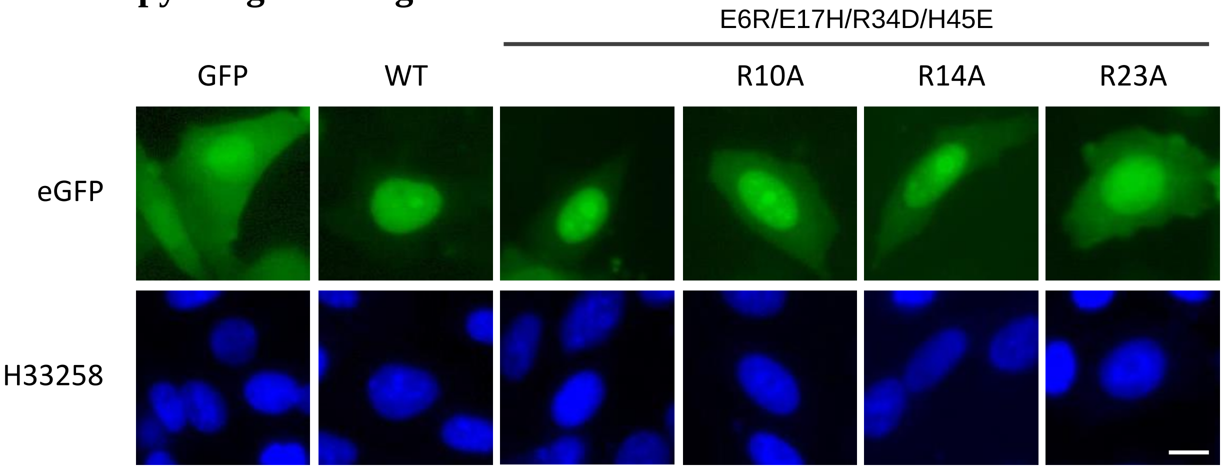

Microscopy images for Fig 3E

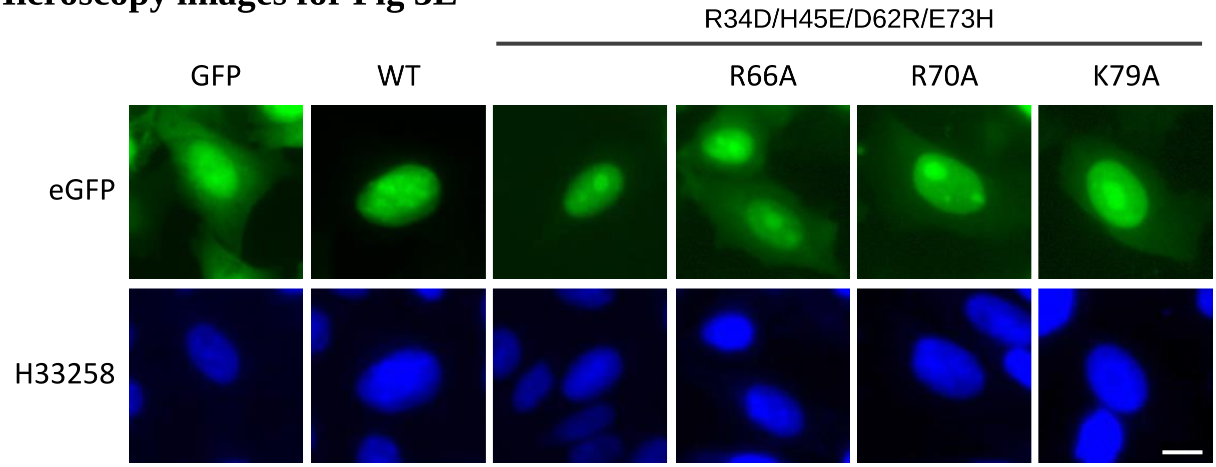

Microscopy images for Fig 4A

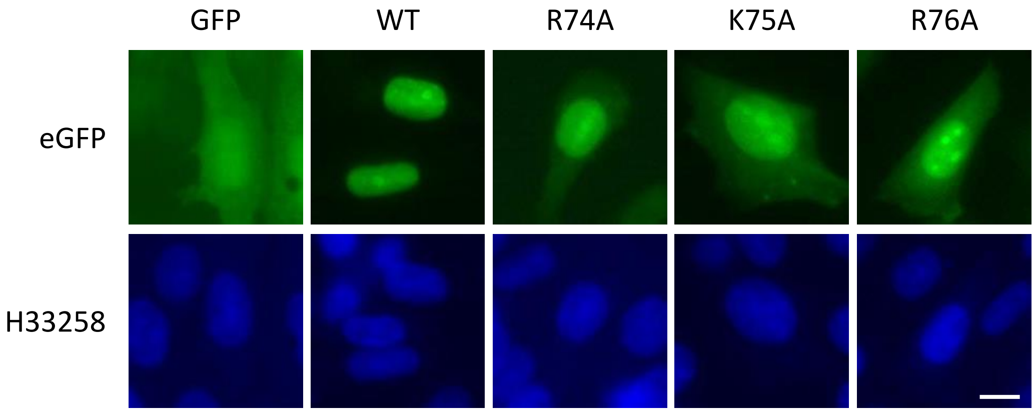

Microscopy images for Fig 4B

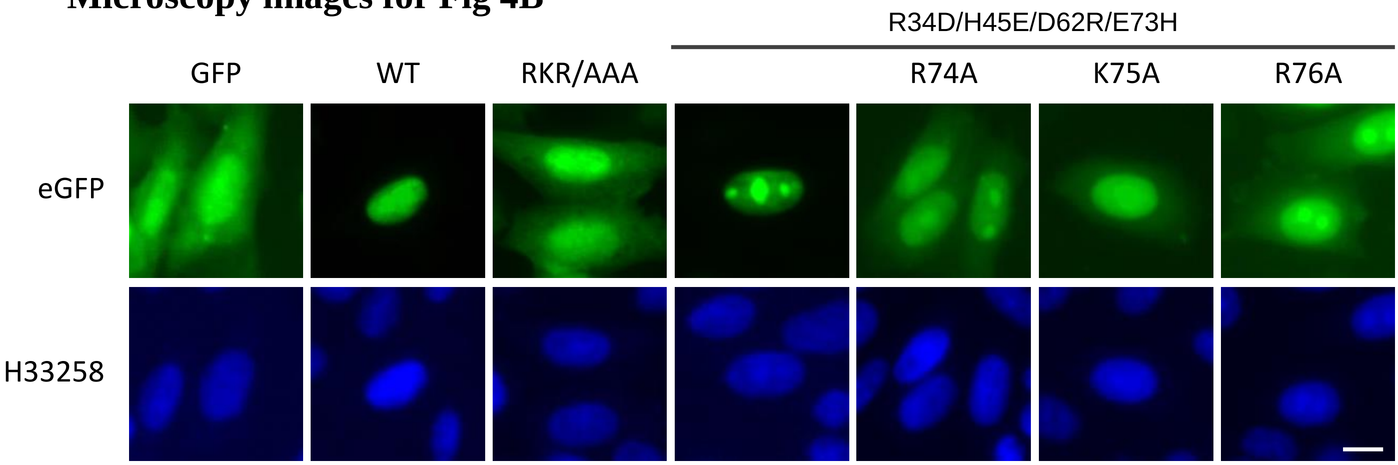

Microscopy images for Fig 4C

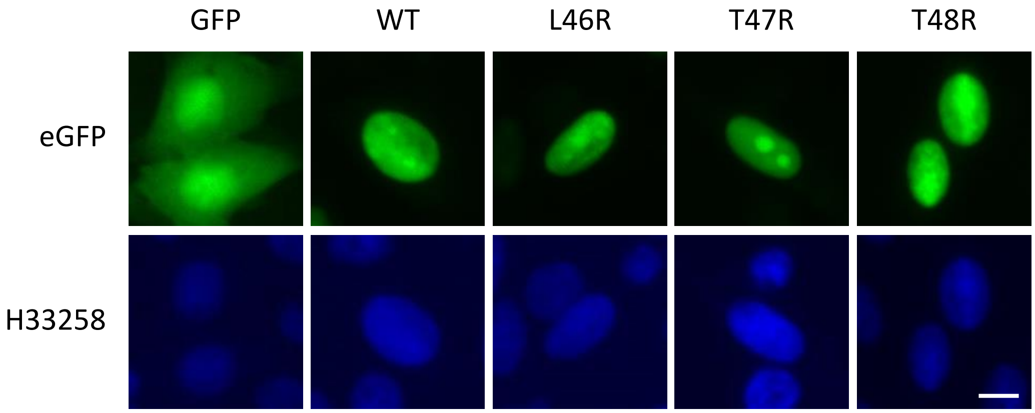

Microscopy images for Fig 6C

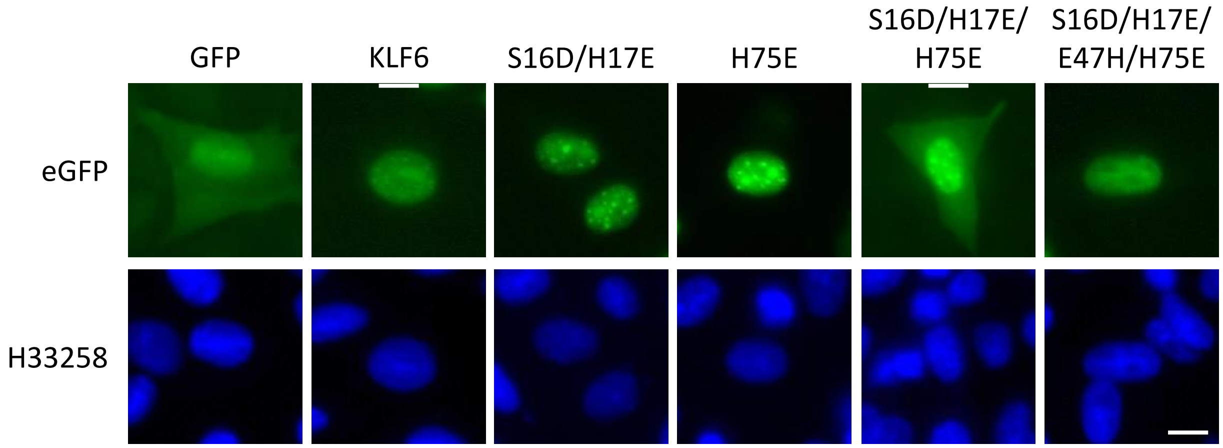

Microscopy images for Fig 6D

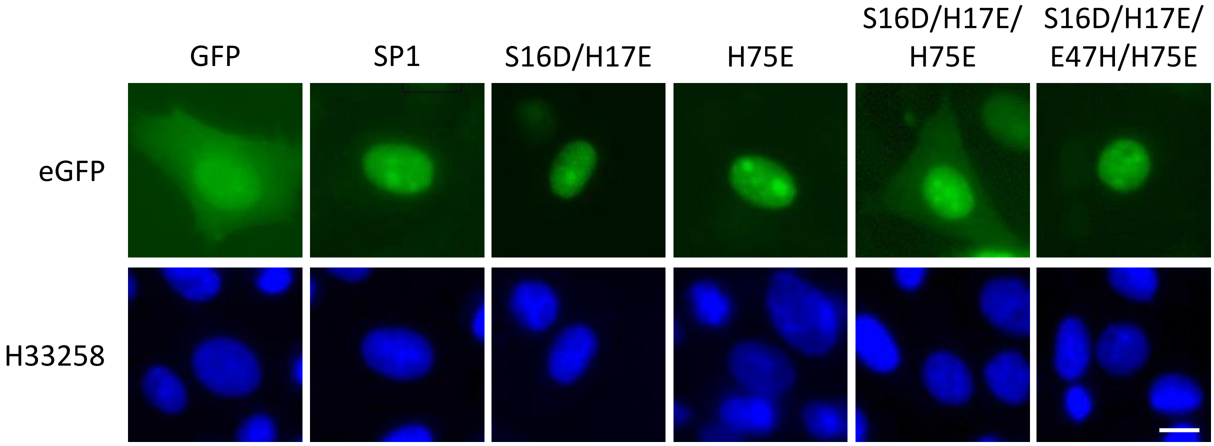

Supplement: S1 Fig — The nuclei were stained with DAPI (blue). Scale bars, 10 μm. (PDF) [file pone.0191971.s003.pdf]
