# Supplementary figures and images for: The conserved basic residues and the charged amino acid residues at the α-helix of the zinc finger motif regulate the nuclear transport activity of triple C2H2 zinc finger proteins
Source: PLoS One. 2018 Jan 30;13(1):e0191971. doi: 10.1371/journal.pone.0191971 (PMC5790263; doi:10.1371/journal.pone.0191971)

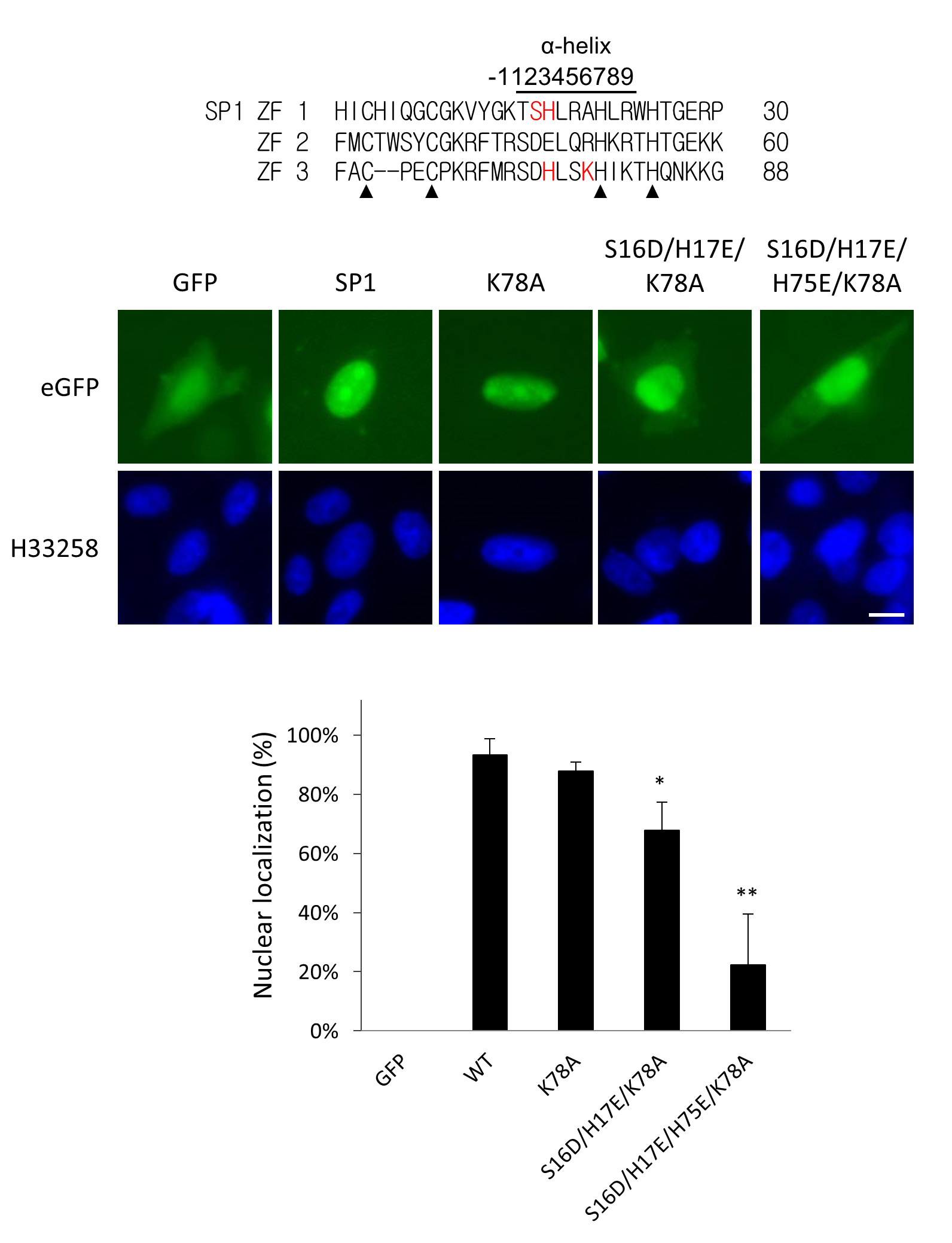

Supplement: S2 Fig — The Lys78 (α6) in the α-helical region of SP1 were mutated to alanine with S16D/H17E or S16D/H17E/H75E. The plasmids were transfected into CHO K1 cells for protein expression. The proportion of cells having the GFP fusion protein entirely localized in the nucleus was determined. Each value represents a mean ± standard deviation of three independent samples. Asterisk indicates significant difference as compared to that of the wild type (WT) fusion protein. *: p < 0.05; **: p < 0.01. SP1 ZF sequences are shown on the top panel with the mutated residues in red. Solid triangles indicate the conserved Cys and His residue in the ZFs. Representative microscopic images are shown in the middle panel. Scale bars, 10 μm. (TIF) [file pone.0191971.s004.tif]

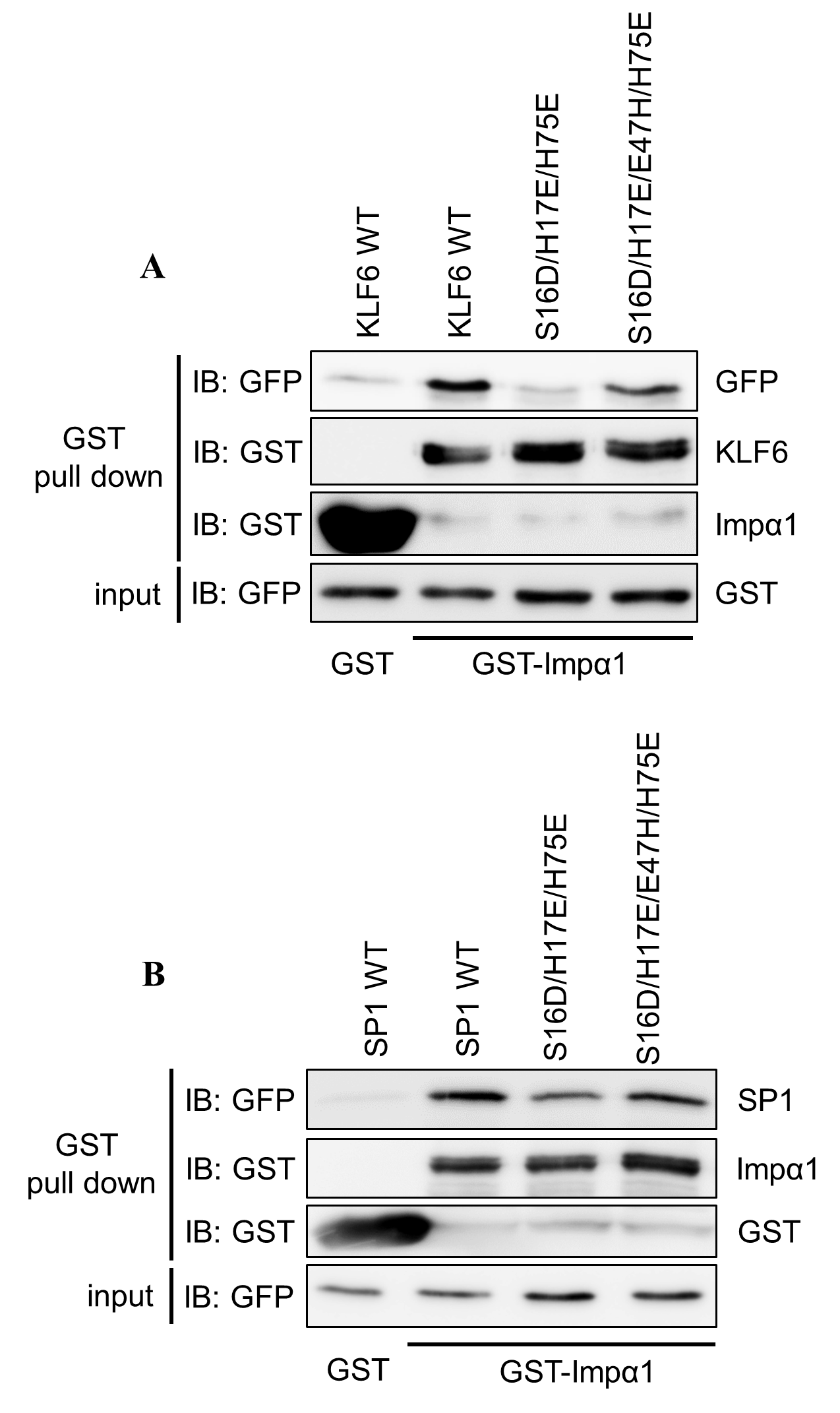

Supplement: S3 Fig — Cell extracts containing wild type and mutated GFP-KLF6 ZFD (A) or GFP-SP1 ZFD (B) fusion protein were incubated with extracts expressing GST-Impα1. The interactions between the fusion proteins and GST-Impα1 were analyzed by the GST pull-down assay. (TIF) [file pone.0191971.s005.TIF]

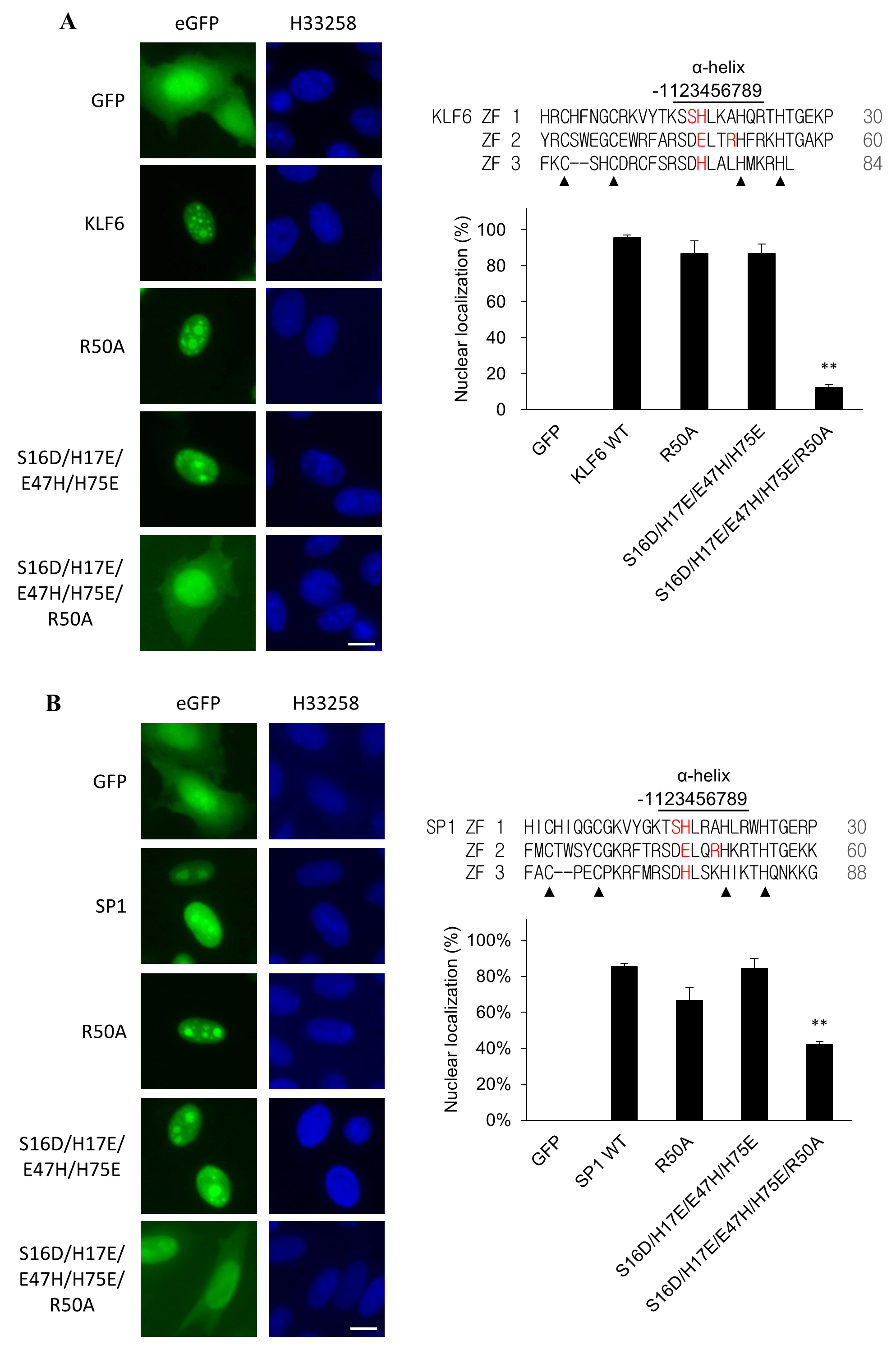

Supplement: S4 Fig — The α2-α3 residues in the α-helical region of KLF6 (A) or SP1 (B) were mutated (S16D/H17E/E47H/H75E) to mimic that of Egr-1 (DE, DH and DE at α2-α3 of ZF1, ZF2 and ZF3, respectively). The basic residue at α6 of the ZF2 was further mutated (R50A). The plasmids were transfected into CHO K1 cells for protein expression. The proportion of cells having the GFP fusion protein entirely localized in the nucleus was determined. Each value represents a mean ± standard deviation of three independent samples. Asterisk indicates significant difference as compared to that of the wild type (WT) fusion protein. **: p < 0.01. The sequence of the KLF6 or SP1 ZFD is shown in the upper panel of each figure with the mutated residues in red. Solid triangles indicate the conserved Cys and His residue in the ZFs. Representative microscopic images are shown in the left panel of each figure. Scale bars, 10 μm. (TIF) [file pone.0191971.s006.TIF]
